# Targeting Fibrosis: From Molecular Mechanisms to Advanced Therapies

**DOI:** 10.1002/advs.202410416

**Published:** 2024-12-12

**Authors:** Xingpeng Di, Ya Li, Jingwen Wei, Tianyue Li, Banghua Liao

**Affiliations:** ^1^ Department of Urology and Institute of Urology West China Hospital Sichuan University Chengdu P.R. China

**Keywords:** clinical trial, fibrosis, mechanism, signaling pathway, therapy

## Abstract

As the final stage of disease‐related tissue injury and repair, fibrosis is characterized by excessive accumulation of the extracellular matrix. Unrestricted accumulation of stromal cells and matrix during fibrosis impairs the structure and function of organs, ultimately leading to organ failure. The major etiology of fibrosis is an injury caused by genetic heterogeneity, trauma, virus infection, alcohol, mechanical stimuli, and drug. Persistent abnormal activation of “quiescent” fibroblasts that interact with or do not interact with the immune system via complicated signaling cascades, in which parenchymal cells are also triggered, is identified as the main mechanism involved in the initiation and progression of fibrosis. Although the mechanisms of fibrosis are still largely unknown, multiple therapeutic strategies targeting identified molecular mechanisms have greatly attenuated fibrotic lesions in clinical trials. In this review, the organ‐specific molecular mechanisms of fibrosis is systematically summarized, including cardiac fibrosis, hepatic fibrosis, renal fibrosis, and pulmonary fibrosis. Some important signaling pathways associated with fibrosis are also introduced. Finally, the current antifibrotic strategies based on therapeutic targets and clinical trials are discussed. A comprehensive interpretation of the current mechanisms and therapeutic strategies targeting fibrosis will provide the fundamental theoretical basis not only for fibrosis but also for the development of antifibrotic therapies.

## Graphical abstract

This review comprehensively summarizes the advances in research for fibrosis. It emphasizes organ‐specific profibrotic cell types and molecular mechanisms of fibrosis, especially in the kidney, liver, lung, and heart. The authors highlight the advanced antifibrotic small molecule drugs and cellular therapies for fibrosis. These findings have critical implications for further research and clinical translation of medicine.

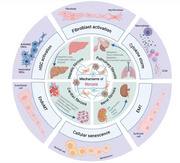

## 1 Introduction

Pathological fibrosis, a process of excessive extracellular matrix (ECM) deposition in the interstitium, is an outcome of abnormal and imbalanced tissue repair rather than a disease.^[^ ^1^ ^]^ The cumulative annual incidence of fibrosis is approximately 4,968 per 100,000 person‐years worldwide, contributing to ∼45% of all deaths in the industrial world.^[^ ^2^ ^]^ Fibrotic disorders accounted for a substantial number of patients with disability‐adjusted life‐years (DALYs) in 2019 worldwide.^[^ ^3^ ^]^

In general, genetic heterogeneity, aging, virus infection, alcohol, diabetes, abnormal biomechanical stimuli, radiation, and environmental or invading foreign bodies determine the progression from tissue repair to pathological fibrosis.^[^ ^4^, ^5^, ^6^ ^]^ Although the aetiologies and mechanisms of these fibrotic disorders are diverse, the major factors contributing to their pathogenesis are common. For example, transforming growth factor β (TGF‐β) signaling is critical in nearly all types of fibrosis.^[^ ^7^ ^]^ These factors establish the relationship between inflammation and ECM remodeling, which sheds light on the investigation of antifibrotic therapies.

During tissue repair, injured sites activate effector cells, such as tissue‐resident fibroblasts and immunocytes to increase the contractility of tissue. Inflammatory mediators are synthesized, and a fibrotic niche is formed. The production of the ECM facilitates wound healing, which may result in pathological fibrosis. In addition, some parenchymal cells activated by injury also engage in fibrogenic responses, such as the activation of hepatic stellate cells (HSCs) in hepatic fibrosis.^[^ ^8^ ^]^ Although this fibrogenic response may be beneficial and critical in the early stage of tissue repair, prolonged accumulation of ECM (e.g., collagen and fibronectin) destroys the structure of the parenchymal capsule and leads to cellular dysfunction and organ failure.^[^ ^9^ ^]^

**Figure 1:**
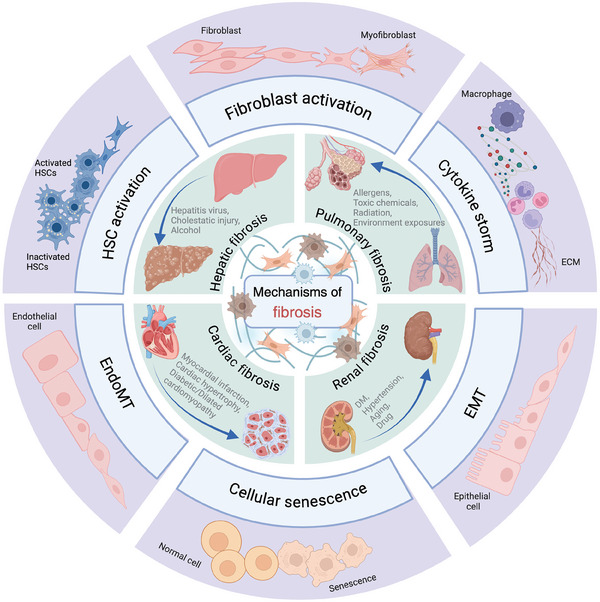
Overview of the aetiologies and mechanisms of fibrosis in kidney, liver, lung, and heart. ECM, extracellular matrix; EMT, epithelial‐to‐mesenchymal transformation; EndoMT, endothelial‐to‐mesenchymal transformation; DM, diabetes mellitus; HSC, hepatic stellate cell. Created with Biorender.com.

Fibrotic disorders, include but are not limited to, pulmonary fibrosis, systemic sclerosis, cardiac fibrosis, liver cirrhosis, and renal fibrosis^[^ ^10^ ^]^ (**Figure** **1**). Some pathophysiological processes that are not easily identified are accompanied by fibrotic disorders, such as cancer behaviors and macular degeneration.^[^ ^11^, ^12^ ^]^ Fibrosis facilitates the progression of diseases, leading to life‐threatening outcomes. In general, this “out‐of‐control” wound healing response involves sophisticated signal transduction involving activities and interactions within and among different cell types, such as fibroblasts, immunocytes, HSCs, endothelial cells, and epithelial cells. Advanced single‐cell RNA sequencing (scRNA‐seq) helps to identify more detailed tissue‐ and organ‐specific cell subtypes, especially individual tissue‐resident fibroblast subtypes related to pathological fibrosis. Researchers have established an atlas of fibroblasts through the integration of scRNA‐seq data across healthy and diseased organs.^[^ ^13^ ^]^ Importantly, the communication among these cell types can largely be detected through scRNA‐seq, which provides deep and definite mechanisms of fibrosis.

In the current review, we systematically summarized the molecular mechanisms of pathological fibrosis. We focus mainly on the etiology, epidemiology, mechanisms and advanced therapies for renal fibrosis, hepatic fibrosis, pulmonary fibrosis, and cardiac fibrosis.

## 2 A history Overview of Fibrosis

**Figure 2:**
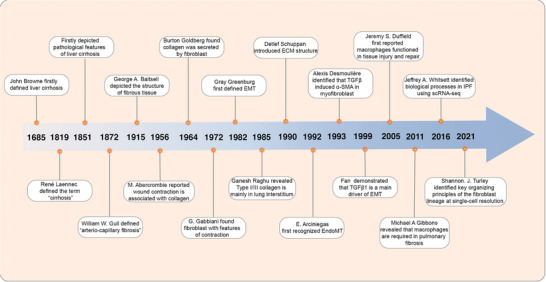
Timeline of the milestones in the investigation of fibrosis over the past 300 years. ECM, extracellular matrix; EMT, epithelial‐to‐mesenchymal transformation; EndoMT, endothelial‐to‐mesenchymal transformation; scRNA‐seq, single‐cell RNA sequencing.

More than 300 years ago, researchers reported abnormal pathological changes in organs, which could be the first sight of fibrosis. In 1685, John Browne first defined liver cirrhosis as the “liver appearing glandulous”^[^ ^14^ ^]^ (**Figure** **2**). He also provided the first definition of necrotizing pancreatitis in 1684. In 1819, René Laennec was the person who defined the term “cirrhosis”, which means tawny or brownish yellow but not simply yellow.^[^ ^15^ ^]^ This type of cirrhosis initially depends on the deposition of morbid substances in the liver. In 1851, Hawley JS reported a case with a liver specimen by Professor Flint, which is a typical pathological depiction of liver cirrhosis.^[^ ^16^ ^]^ In this case, researchers and clinicians focused on liver diseases caused by obstruction of the portal system. Similarly, Corrigan first defined lung cirrhosis as “the fibrous covering in the lung, cellular tissue, and elastic lining of tubes elevate the contraction of lung tissue”.^[^ ^17^ ^]^ In 1852, Bristowe reported the first pathological anatomy of lung cirrhosis.^[^ ^18^ ^]^ The lung tissue sections revealed extensive consolidation nearly everywhere, accompanied by dilation of the tubes. Meanwhile, Richard Quain first described cardiac fibrosis as “fatty diseases of the heart”.^[^ ^19^ ^]^ The peculiar fatty condition of the heart was found external to the heart's fibers. The abnormal tissue is more like a complex consisting of fibrous tissue, fat cells, and muscular fibers, which was initially described as fibrous degeneration by Williams. W.H. Dickinson defined the two essentially distinct conditions of kidney disease in 1859.^[^ ^20^ ^]^ There are two distinct stages of “kidney tubular disease”. The first stage is enlargement, and the second stage is diminution. The second stage is the late stage of the kidney, which is rarely reached. Damage to tubes and superficial nerves leads to the fibrous nature of the kidney. Although researchers have not defined and categorized these pathological changes into unified diseases, these findings are all important prerequisites for further investigations of fibrotic diseases.

Upon the discovery of kidney disease, William W. Gull first defined hyaline‐fibroid formation in arterioles and capillaries as “arterio‐capillary fibrosis” in 1872.^[^ ^21^ ^]^ Afterwards, Dyce Duckworth reported two cases of “hypertrophic fibrosis of the liver”.^[^ ^22^ ^]^ He believed that the enlargement of the liver was not infiltrated by new growth of the organ but rather by biliary cirrhosis or fibrosis.

The discovery and definition of fibrosis have attracted increased attention. Researchers have begun to investigate the features of fibrosis. In 1915, George A. Baitsell illustrated the origin and structure of fibrous tissue in adult frog tissues.^[^ ^23^ ^]^ The repair of continuous or repetitive injury to tissue is related to fibrosis. In 1956, M. Abercrombie first reported that wound contraction is associated with collagen formation in an animal model.^[^ ^24^ ^]^ In 1985, Ganesh Raghu compared the components of the ECM in normal and fibrotic human lungs. The results revealed that type I collagen (characterized in 1969) and type III collagen accumulate mainly in the lung interstitium of patients with pulmonary fibrosis.^[^ ^25^ ^]^ He also reported that type III collagen was the main component in the alveolar septa and interstitium in the early stage, whereas type I collagen was predominant in the late stage of fibrosis. Later, in 1990, Detlef Schuppan systematically introduced the ECM structure in normal and fibrotic livers. The collagens (e.g., type I and III collagen) represent only 5–10% of the total protein in the liver, which accounts for 50% or more of the total proteins in fibrotic liver. In addition, glycoproteins (e.g., fibronectin) accumulate sharply in hepatic fibrosis.^[^ ^26^ ^]^

However, there was still no consensus on the sources of collagens in the 1950s. In 1964, Burton Goldberg reported that collagen was secreted by an established mouse fibroblast line.^[^ ^27^ ^]^ Through electron microscopy, they reported that collagen appeared to be synthesized in the endoplasmic reticulum (ER) and transported outside the cell membrane, thereby forming fibrils of two‐diameter distributions. Similarly, Albert K. Harris demonstrated that greater traction force in untransformed fibroblasts was associated with ECM rearrangement.^[^ ^28^ ^]^ In 1972, G. Gabbiani reported that contracting granulation tissues contain fibroblasts with features from smooth muscle during wound contraction, which is the early depiction of myofibroblasts.^[^ ^29^ ^]^ For example, they reported that the fibrillar system in activated fibroblasts is similar to bundles of parallel fibrils resembling those of smooth muscle cells rather than normal fibroblasts. However, although these myofibroblasts express α‐smooth muscle actin (α‐SMA), they still differentiate from fibroblasts rather than muscle cells.^[^ ^30^ ^]^ Rapidly, more research on wound healing has focused on the differentiation and contraction of localized fibroblasts.^[^ ^31^ ^]^ Importantly, in 1993, Alexis Desmoulière and colleagues identified the critical role of TGF‐β1 in inducing myofibroblast differentiation during wound healing.^[^ ^32^ ^]^ Notably, TGF‐β1, rather than other cytokines and growth factors (e.g., platelet‐derived growth factor [PDGF] and tumor necrosis factor‐α [TNFα]), can induce α‐SMA in myofibroblasts. Since then, researchers and scientists have recognized the important role of fibroblasts in wound healing and fibrosis.

In addition to fibroblasts, other cell types, such as parenchymal cells and immunocytes, also play crucial roles in fibrosis. The phenotype, shape, and polarity of epithelial cells are reported to be affected by environmental conditions. In 1982, Gray Greenburg first demonstrated that specific stimulation promotes the transformation of epithelial cells to a mesenchymal phenotype, which is referred to as epithelial‐to‐mesenchymal transformation (EMT).^[^ ^33^ ^]^ EMT was originally regarded as an early stage in embryogenesis, conferring migration in the formation of mesoderm and enabling the migration of primitive neuroepithelia to neural crest cells.^[^ ^33^ ^]^ In 2002, Masayuki Iwano and colleagues reported that fibroblasts not only arise from primitive mesenchymal cells that survive embryonic development, but also differentiate from mesenchymal cells that express fibroblast‐specific protein‐1 (FSP1).^[^ ^34^ ^]^ These findings suggest that approximately 36% of novel fibroblasts are derived from EMT in kidney fibrogenesis, whereas only approximately 15% are derived from the bone marrow. In 1999, Fan et al. demonstrated that TGF‐β1 is a main driver of EMT‐associated tubulointerstitial fibrosis.^[^ ^35^ ^]^ EGF, IGF‐II, or fibroblast growth factor‐2 (FGF2) facilitate the EMT process.^[^ ^36^, ^37^, ^38^, ^39^ ^]^

Additionally, in 1992, E. Arciniegas et al. reported that bovine aortic endothelial cells exposed to TGF‐β1 could be differentiated into a smooth muscle‐like phenotype,^[^ ^40^ ^]^ which was the earliest recognition of endothelial‐to‐mesenchymal transformation (EndoMT). Leonard M. Eisenberg formally recognized EndoMT during embryonic heart development, in which endothelial cells are bipotential stem cells that can be transdifferentiated into endocardial mesenchymal cells.^[^ ^41^ ^]^ In 2007, Zeisberg and collaborators demonstrated that EndoMT plays an important role in cardiac fibrosis.^[^ ^42^ ^]^ In fibrotic kidneys, the percentages of endothelial marker cluster of differentiation 31 (CD31)^+^, FSP1^+^, and α‐SMA^+^ fibroblasts account for 30–50% of the total population.^[^ ^43^ ^]^ Researchers subsequently focused more on the mechanisms involved in the relationship between EndoMT and organ fibrosis.

During fibrosis, the wound recruits immunocytes to trigger an immune response. In 1975, S. J. Leibovich demonstrated that macrophages are the principal cell type involved in wound debridement clearance.^[^ ^44^ ^]^ Additionally, fibroblasts are triggered by macrophages in this process. In 2005, Jeremy S. Duffield provided the first clear evidence that macrophages have potential for both injury‐inducing and repair‐promoting functions.^[^ ^45^ ^]^ In hepatic fibrosis, the depletion of macrophages leads to the alleviation of both scarring and fibroblast activation. In 2011, Michael A Gibbons et al. demonstrated that macrophages are required for bleomycin‐induced pulmonary fibrosis.^[^ ^46^ ^]^ In 1989, Khalil N reported for the first time that macrophages can produce TGF‐β1 and promote the synthesis of ECM in a bleomycin‐induced pulmonary fibrosis rat model.^[^ ^47^ ^]^ Similarly, Lynne A Murray and colleagues reported that the production of TGF‐β1 by macrophages drove pulmonary fibrosis.^[^ ^48^ ^]^

Researchers are not only clarifying the mechanisms of fibrosis in‐depth but also investigating sufficient therapies (e.g., cellular therapy) targeting fibrosis. Previous studies revealed the capacity of mesenchymal stem cells (MSCs) to secrete a wide range of exosomes that could be exploited as potential therapies for fibrosis. MSC engraftment has shown ideal efficacy in a bleomycin‐induced pulmonary fibrosis mouse model.^[^ ^49^ ^]^ In 2004, Isao Sakaida revealed the transplantation of MSCs alleviated carbon tetrachloride (CCL_4_)‐induced hepatic fibrosis in mice.^[^ ^50^ ^]^ In 2005, Florian demonstrated that MSCs are a potential therapy targeting renal failure.^[^ ^51^ ^]^ Although the mechanisms of fibrosis have not been clarified yet, cellular therapy is still an alternative therapeutic strategy for fibrosis.

In 2016, Jeffrey A. Whitsett et al. identified subtypes of epithelial cells and biological processes involved in pulmonary fibrosis using scRNA‐seq.^[^ ^52^ ^]^ In 2021, Shannon. J. Turley provided major organizing principles of the fibroblast lineage at single‐cell resolution.^[^ ^13^ ^]^ This study constructed a fibroblast atlas that paves the way for precise therapies targeting fibrosis. In summary, research on fibrosis has lasted more than 300 years, and great progress has been made in understanding the mechanisms and therapies targeting fibrosis. Currently, the mechanisms of fibrosis are more specific, clearer, and more in depth, providing potential for the exploration of individualized and sufficient therapies targeting fibrosis.

## 3 Molecular Mechanisms of Organ‐Specific Fibrosis

Although fibrosis is a common outcome of severe and prolonged organ injury, the cell populations and molecular mechanisms contributing to fibrogenesis in different organs and tissues are not the same (**Table** **1**). The primary cells involved in ECM production and immunopathology in organ‐specific fibrosis are peculiar and need to be clarified.

**Table 1:** Potential primary cells and mechanisms involved in fibrosis of kidney, liver, lung, and heart.

Organ	Cell type	Mechanisms	Reference
Kidney	TECs	EMT to myofibroblast	[34, 53]
	Endothelial cells	EndoMT to myofibroblast	[54]
	Monocytes/macrophages	i. Cytokine production	[55]
		ii. MMT to myofibroblast	[56]
	Bone marrow‐derived fibrocytes	Recruiment and transdifferentiation to myofibroblast	[57]
	Resident fibroblasts	Proliferation and transdifferentiation to myofibroblast	[57]
	Pericytes	Transdifferentiation to myofibroblast	[58]
	Podocytes	EMT to myofibroblast	[59]
	Gli^+^ perivascular MSCs	Proliferation and transdifferentiation to myofibroblast	[60]
	MSCs	TGF‐β1 dependent differentiation to myofibroblast	[57]
	REP cells	i. Dedifferentiation of REP cells leads to loss of Epo synthesis	[61]
		ii. Reduced energy consumption of collapsed nephrons causes hyperoxia and inhibit Epo production	[62]
		iii. HIF signaling‐induced transdifferentiation from REP cells to myofibroblasts	[63]
Liver	Pericytes (e.g., HSCs)	Transdifferentiation to myofibroblast	[64, 65]
	Portal fibroblasts	Transdifferentiation to myofibroblast	[64, 66]
	Resident fibroblasts	Proliferation and transdifferentiation to myofibroblast	[67]
	Vascular smooth muscle cells	Transdifferentiation to myofibroblast	[67]
	Bone marrow‐derived fibrocytes	Recruiment and transdifferentiation to myofibroblast	[64]
	Hepatocytes	EMT to myofibroblast	[68]
	Biliary epithelial cells	EMT to myofibroblast	[69]
	Mesothelial cells	Mesothelial–mesenchymal transition	[70]
	Gli^+^ perivascular MSCs	Proliferation and transdifferentiation to myofibroblast	[60]
	Sinusoidal endothelial cells	i. Cytokine production	[71]
		ii. Defenestration and capillarization	[72, 73]
	Kupffer cells	i. Cytokine production	[74]
		ii. Activate HSCs	[75]
		iii. Regression of hepatic fibrosis	[76]
Lung	Resident fibroblasts	Proliferation and transdifferentiation to myofibroblast	[77]
	Bone marrow‐derived fibrocytes	Recruiment and transdifferentiation to myofibroblast	[78]
	Pericytes	Transdifferentiation to myofibroblast	[79]
	AECs	i. EMT to myofibroblast	[80]
		ii. Cytokine and chemokine production	[81]
	Endothelial cells	EndoMT to myofibroblast	[79]
	Gli^+^ perivascular MSCs	Proliferation and transdifferentiation to myofibroblast	[60]
	Mesothelial cells	Mesothelial–mesenchymal transition	[79]
	Macrophages	i. Cytokine production	[82, 83]
		ii. Polarization to profibrotic phase	[84]
	Other Immunocytes (Dendritic cell, neutrophil, eosinophil, etc.)	i. Cytokine and chemokine production	[85]
		ii. Cytokine storm formation in SARS‐CoV‐2 infected lung	[5]
Heart	Cardiac fibroblasts	i. Cytokine and chemokine production	[86]
		ii. Proliferation and transdifferentiation to myofibroblast	[87]
	Cardiomyocytes	Cytokine and chemokine production	[88, 89]
	Bone marrow‐derived fibrocytes	Recruiment and transdifferentiation to myofibroblast	[90]
	Endothelial and vascular smooth muscle cells	Transdifferentiation to myofibroblast	[87, 91]
	Gli^+^ perivascular MSCs	Proliferation and transdifferentiation to myofibroblast	[60]
	Epicardial cells	EMT to myofibroblast	[92]
	Macrophages	i. Polarization to inflammation/profibrotic phase	[93]
		ii. Exosome secretion	[93, 94]
	Other immunocytes	Cytokine and chemokine production	[95]

AECs, alveolar epithelial cells; EMT, epithelial‐to‐mesenchymal transformation; EndoMT, endothelial‐to‐mesenchymal transformation; Epo, erythropoietin; HSCs, hepatic stellate cells; MMT, macrophage‐to‐mesenchymal transformation; MSCs, mesenchymal stem cells; REP, renal Epo‐producing; SARS‐CoV‐2, severe acute respiratory syndrome coronavirus 2; TECs, tubular epithelial cells; TGF‐β, transforming growth factor β.

### 3.1 Renal Fibrosis

Renal fibrosis is a severe and irreversible outcome of nearly all types of chronic kidney disease (CKD), and is characterized by pathological ECM deposition in the interstitial space and within the glomerular capillary wall.^[^ ^96^ ^]^ Renal fibrosis indicates renal dysfunction and poor prognosis. CKD is defined as persistent abnormalities in kidney structure and function (i.e., estimated glomerular filtration rate (eGFR) < 60 mL min^−1^ per 1.73 m^2^ or urinary albumin‐creatine ratio (ACR) ≥30 mg g^−1^) lasting for >3 months, which is the 16th leading cause of death globally.^[^ ^97^ ^]^ However, only <5% of CKD patients are diagnosed in a timely manner. The etiologies include diabetes mellitus, hypertension, obesity and aging. These risk factors ultimately lead to irreversible nephron loss, reduced renal regenerative capacity, metabolic alterations, oxidative stress, end‐stage renal disease, and/or premature death.^[^ ^98^ ^]^

**Figure 3:**
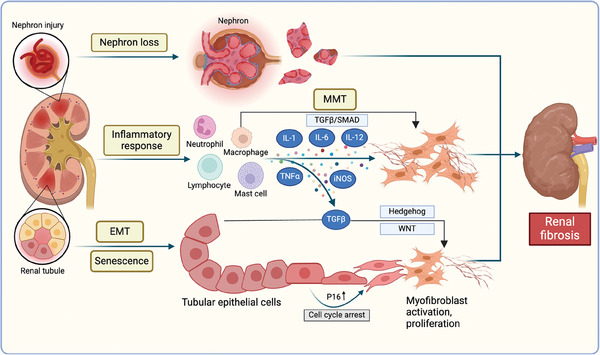
The mechanisms from kidney injury to fibrosis and the origin of myofibroblasts. The nephrons and renal tubules are shown. Kidney injury induces the generation and accumulation of ECM that replaces the functional nephron unit, leading to nephron loss. Moreover, injury to and transdifferentiation of renal tubules also occur during renal fibrosis. In addition to resident fibroblasts in the interstitial space of the kidney, injured renal tubular epithelial cells (TECs), pericytes, perivascular fibroblasts, and macrophages are also potential origins of myofibroblasts. In response to inflammation, the recruited neutrophils, lymphocytes, mast cells, and macrophages secrete IL‐1, IL‐6, IL‐12, TNF‐α, and iNOS to directly activate resident fibroblasts into myofibroblasts. Macrophage‐to‐mesenchymal transformation (MMT) also occurs under the stimulation of TGF‐β/SMAD signaling. In addition, TECs are transformed into activated myofibroblasts through epithelial‐to‐mesenchymal transformation (EMT) and cellular senescence. Created with Biorender.com.

Renal fibrosis is generally a wound‐healing process that occurs in response to kidney injury and prevents further damage to structural and functional integrity (**Figure** **3**). However, severe or uncontrolled repeated injury to the kidney leads to pathological deposition of ECM in the interstitial space.^[^ ^99^ ^]^ As a result, nephron loss occurs due to podocyte loss and replacement by the ECM (termed glomerulosclerosis), tubular cell injury and transdifferentiation, and subsequent tubulointerstitial fibrosis.^[^ ^100^, ^101^ ^]^ scRNA‐seq results revealed that the fibrotic niche in the human fibrotic kidney mainly includes mesenchymal cells, immunocytes, and tubular epithelial cells (TECs).^[^ ^102^ ^]^ RNA‐seq also provides evidence for multiple subpopulations of renal fibroblasts, which can help distinguish fibrosis‐related fibroblasts.

#### 3.1.1 Origin, Heterogeneity, and Role of Myofibroblasts in Renal Fibrosis

The number of myofibroblasts is low in normal kidneys but especially high in fibrotic kidneys.^[^ ^103^ ^]^ However, the origin and heterogeneity of myofibroblasts have not yet been clarified. Stromal cells, also classified as resident mesenchymal cells, reside in the interstitial space of the kidney and are considered the common origin of myofibroblasts. Stromal cells include fibroblasts, MSCs, and pericytes.^[^ ^104^ ^]^ Local resident fibroblasts and bone marrow‐derived fibrocytes are major origins of myofibroblasts. Proliferation is the main feature of conversion between local resident fibroblasts and myofibroblasts (50%), whereas migration and differentiation are the main approaches for the conversion of bone marrow‐derived fibrocytes to myofibroblasts (35%).

In addition, MSCs also contribute to myofibroblast recruitment in renal fibrosis via TGF‐β signaling.^[^ ^57^ ^]^ TGF‐β type II receptor (TGF‐βR2) is the transducer of TGF‐β signaling, which recruits TGF‐βR1 and phosphorylates Drosophila mothers against decapentaplegic 2/3 (SMAD2/3). In TGF‐βR2; α‐SMA‐Cre transgenic mice, α‐SMA expression and fibroblast differentiation are attenuated in renal fibrosis.^[^ ^57^ ^]^ Compared with those in control mice, a 56% reduction in myofibroblasts was found in transgenic mice with renal fibrosis. However, α‐SMA levels and the accumulation of ECM are not changed in TGF‐βR2 transgenic mice.^[^ ^105^ ^]^ One possible reason could be that the technical restriction of the cre/loxP system limits the outcome for all subtypes of fibroblasts. In the former study, the researchers chose α‐SMA as a marker for fibroblasts, whereas the latter selected PDGF receptor β (PDGFRβ) as a marker. Another possible explanation could be that the expression of α‐SMA and myofibroblast differentiation in PDGFRβ^+^ cells is not solely regulated by the TGF‐β signaling pathway. In general, α‐SMA is a characteristic marker of myofibroblasts. However, not all active fibroblasts express α‐SMA.^[^ ^106^ ^]^ Lin et al. demonstrated that most collagen‐positive cells co‐stained with α‐SMA in collagen‐GFP transgenic mice. Nevertheless, 25% of α‐SMA^+^ cells in the interstitial space do not express collagen.^[^ ^107^ ^]^ There are still 1% of type I collagen‐producing interstitial cells that do not express α‐SMA. Advanced evidence suggests that α‐SMA expression cannot be used to distinguish myofibroblasts from other cell types accurately. Single‐cell technologies have provided detailed depictions of the heterogeneity of cell types.^[^ ^108^ ^]^

Christoph Kuppe and colleagues mapped all ECM‐producing cell types, revealing distinct subpopulations of fibroblasts, which are major sources of myofibroblasts during renal fibrosis.^[^ ^102^ ^]^ They reported that the ECM overproduced in human renal fibrosis is attributed mainly to mesenchymal cells rather than dedifferentiated TECs. Pdgfra^+^/Pdgfrb^+^ dual‐positive fibroblasts are the main source of myofibroblasts in renal fibrosis. Additionally, Notch3^+^/RGS5^+^/Pdgfra^−^ pericytes, Meg3^+^/Pdgfra^+^ fibroblasts, and Colec11^+^/Cxcl12^+^ fibroblasts are three major sources of myofibroblasts in human fibrotic kidneys. However, whether pericytes contribute to renal fibrosis is still controversial. Valerie S. LeBleu et al. demonstrated that specific deletion of pericytes does not alleviate renal fibrosis or the recruitment of myofibroblasts in a unilateral ureteral obstruction (UUO) mouse model.^[^ ^57^ ^]^ However, some studies hold a different view of the potential for differentiation between pericytes and myofibroblasts in the same UUO mouse model.^[^ ^58^, ^107^ ^]^ They reported that α‐SMA^−^ interstitial pericytes differentiated into α‐SMA^+^ myofibroblasts during fibrosis.

In addition to the traditional origins of myofibroblasts, injured TECs are also important precursors of myofibroblasts in renal fibrosis.^[^ ^34^, ^53^ ^]^ However, recent genetic lineage analyses demonstrated that epithelial cells cannot be transformed into myofibroblasts through EMT.^[^ ^109^ ^]^ Rather, pericytes and perivascular fibroblasts may be the origin of myofibroblasts.^[^ ^58^, ^60^ ^]^ For example, Ivica Grgic and colleagues labeled all renal epithelial cells via two transgenic mouse models and were unable to identify genetically labeled cells in the interstitial space within the α‐SMA^+^ myofibroblasts. Li et al. generated YFP‐tagged renal epithelium in a UUO mouse model.^[^ ^110^ ^]^ However, no YFP‐labeled myofibroblasts were detected in the interstitial space. Furthermore, they carried out more experiments in which proximal tubular cells were labeled with Texas‐Red‐conjugated dextran in a UUO mouse model. Similarly, no dextran‐labeled myofibroblasts were found in the interstitial space. These findings reveal that mature renal TECs do not convert to myofibroblasts during renal fibrosis in the UUO model. However, the low absorption of dextran in some cell types may affect the performance of cell tracking.

Erythrocytes, which are regulated by kidney‐derived erythropoietin (Epo) under either normal status or reduced O_2_ saturation conditions, are vehicles for O_2_ transport. In adults, kidney‐derived Epo accounts for 90% of the total Epo level in the body.^[^ ^111^ ^]^ Recent studies have demonstrated that the secretion of Epo by renal EPO‐producing (REP) cells contributes to the progression of renal fibrosis.^[^ ^62^ ^]^ REP cells constitute a unique subpopulation of tubulointerstitial cells located in the poor O_2_‐saturated corticomedullary border,^[^ ^112^ ^]^ with characteristics of fibroblasts, pericytes, and neurons. In addition, a subpopulation of perivascular cells in the kidney can also secrete Epo.^[^ ^113^ ^]^ Current hypotheses regarding the function of Epo in CKD are contradictory. One hypothesis revealed that the dedifferentiation of REP cells might lead to loss of Epo synthesis due to the progression of renal fibrosis.^[^ ^61^ ^]^ Another study demonstrated that the reduced energy consumption of collapsed nephrons might cause hyperoxia and inhibit Epo production.^[^ ^62^ ^]^ The microenvironment does not support EPO synthesis in REP cells but also does not influence the function of REP cells. Epo production can be restored by anti‐inflammatory treatment in a UUO mouse model.^[^ ^114^ ^]^ Concurrently, REP cells can transform into myofibroblasts.^[^ ^115^ ^]^ The conversion of REP cells into myofibroblasts and loss of the capacity for the production of Epo, which is regulated by the HIF signaling pathway, are major causes of renal anemia and fibrosis.^[^ ^63^, ^116^ ^]^

#### 3.1.2 Tubular Epithelial Cell Injury and Transformation in Renal Fibrosis

The tubular epithelium is metabolically active and has abundant mitochondria for transportation, which are inversely vulnerable to external injury. Under injury or other abnormal conditions, the renal tubular epithelium loses cell contacts and polarization, leading to functional damage.^[^ ^98^ ^]^ TECs are major cell components of renal parenchyma. Regenerative and repair processes are initiated to protect the properties and functions of TECs. However, uncontrolled tubular damage exceeds the ability of self‐repair, leading to renal fibrosis.

In tissue injury, inflammatory cells (e.g., lymphocytes, monocytes/macrophages, dendritic cells, and mast cells) are recruited to injured sites.^[^ ^117^ ^]^ These activated immunocytes produce profibrogenic cytokines and growth factors and trigger renal fibrosis.^[^ ^55^, ^118^ ^]^ For example, proinflammatory monocytes/macrophages (M1) mediate tubular injury through the production of interleukin 6 (IL‐6), TNFα, IL‐12, and nitric oxide (NO) by iNOS.^[^ ^118^ ^]^ TNFα reportedly promotes tubular apoptosis after kidney injury. Profibrotic macrophages promote wound healing by secreting TGF‐β to activate myofibroblasts and change matrix metalloproteinase (MMP)‐mediated ECM homeostasis.^[^ ^119^ ^]^ As a result, inflammatory responses mediate fibrosis in a paracrine manner, through which profibrotic cytokines act on tubular cells to facilitate fibrosis. An increase in profibrotic cytokines activates ECM‐producing cells (e.g., fibroblasts, TECs, macrophages, and pericytes), promoting the accumulation of a large amount of ECM in the renal interstitial space.

Increasing evidence has demonstrated that EMT contributes directly to the transformation of myofibroblasts during fibrogenesis. EMT has long been considered a source of myofibroblast that contributes to renal fibrosis. Chronic kidney injury changes the local distribution of cytokines and promotes dedifferentiation of epithelial cells, leading to transdifferentiation into myofibroblasts.^[^ ^106^ ^]^ The injured local tubulointerstitial microenvironment reshapes the mesenchymal cell phenotype.^[^ ^120^ ^]^ TGF‐β is a principal regulator of EMT and triggers the proliferation and activation of tissue‐resident fibroblasts. During the EMT process, the transcription factors encoding snail family zinc finger 1 (Snail1) and Twist are two major regulators. M Teresa Grande and colleagues demonstrated that injury‐induced reactivation of Snail1, which promotes partial EMT in mouse renal TECs, is required for the progression of renal fibrosis.^[^ ^121^ ^]^ Therapies targeting Snail1 significantly alleviated UUO‐induced renal fibrosis in mice. In addition, Sara Lovisa et al. revealed that transgenic expression of Twist1 (encoding twist family bHLH transcription factor 1) or Snail1 is critical for prolonged TGF‐β‐induced G2 arrest of tubular cells that still reside associated with the basement membrane.^[^ ^122^ ^]^ Furthermore, in the above renal fibrosis mouse model, conditional *Twist* or *Snail* knockout in proximal tubular cells inhibited EMT and alleviated renal fibrosis. Several recent studies have identified potential therapeutic targets that inhibit the EMT of renal TECs, thereby attenuating renal fibrosis.^[^ ^123^ ^]^ However, some recent studies do not support the contributions of TECs to renal interstitial myofibroblasts. Benjamin D. Humphreys and colleagues labeled and fated renal epithelia in a cre/loxP system renal fibrosis mouse model.^[^ ^58^ ^]^ No evidence has indicated the migration of TECs outside the tubular basement membrane. Another study confirmed the role of TGF‐β in interstitial proliferation, tubular autophagy, and fibrosis rather than in the EMT of renal TECs.^[^ ^124^ ^]^ In fact, dedifferentiated renal TECs undergo partial EMT and are still within the tubular basement membrane expressing epithelia‐related genes. Intriguingly, Tsutomu Inoue et al. reported that such disparities may be attributed to different experimental conditions, such as various mouse models, mouse strains, and genetic alterations.^[^ ^125^ ^]^ As a result, UUO and ischemia‐reperfusion nephropathy in SJL mice are prone to EMT. However, the UUO model using C57B/6 and F1 (C57B/6 X SJL) mice, adriamycin nephropathy in 129 mice, and nephrotoxic serum nephritis in SJL mice are EMT‐resistant models (**Table** **2**). Therefore, EMT is involved in renal fibrosis in both disease‐specific and strain‐specific manners in the mouse kidney. Nevertheless, Yizhen Sang et al. reported that the EMT process still occurs in renal tubular cells 10 days after UUO in C57BL/6 mice.^[^ ^126^ ^]^

**Table 2:** Disease‐specific and strain‐specific animal models of renal fibrosis.

Model type	Strain type	Susceptibility to fibrosis	Susceptibility to EMT	Reference
Obstructive kidney disease	C57BL/6 mice	Susceptible		[127]
	BALB/c mice	Resistant		
	SJL mice		Susceptible	[125]
	C57BL/6 mice		Resistant	
Ischemia‐reperfusion injury of kidney	C57BL/6 mice	Susceptible		[128]
	129Sv mice	Susceptible		
	C57BL/6 mice	Susceptible		[129]
	BALB/c mice	Susceptible		
	NIH Swiss mice	Resistant		
	SJL mice		Susceptible	[125]
5/6 nephrectomy‐induced kidney disease	CD‐1 mice	Susceptible		[130]
	129S3 mice	Susceptible		
	C57BL/6 mice	Susceptible		
Adriamycin nephrosis	129 mice		Resistant	[125]
Nephrotoxic serum nephritis	SJL mice		Resistant	[125]
Doxorubincin‐induced kidney disease	Charles Dawley rats	Susceptible		[131]
	Rowett black hooded rats	Resistant		
Cisplatin‐induced kidney disease	FVB/n mice	Susceptible		[132]
	C57BL/6 mice	Resistant		
Streptozotocin‐induced diabetic nephropathy	CD‐1 mice	Susceptible		[133]
	C57BL/6 mice	Resistant		
	129Sv mice	Resistant		
rhBMP‐7‐induced kidney disease	MRL/MpJ^lpr/lpr^ mice	Susceptible		[134]
	Col4A3^−/−^ mice	Susceptible		
	MRL/MpJ mice	Resistant		
BSA‐induced kidney disease	129S1/svlmJ mice	Susceptible		[135]
	C57BL/6J mice	Susceptible		

BSA, bovine serum albumin; rhBMP‐7, recombinant human bone morphogenic protein 7.

### 3.2 Hepatic Fibrosis

Hepatic fibrosis and cirrhosis are outcomes of persistent liver inflammation, which may lead to the development of hepatocellular carcinoma (HCC).^[^ ^136^ ^]^ Hepatic cirrhosis leads to one million deaths per year globally.^[^ ^137^ ^]^ Like other organs, hepatic fibrosis involves the formation and accumulation of the ECM in the space of Disse,^[^ ^1^ ^]^ leading to disruption of normal tissue architecture and impaired organ function. Hepatic fibrosis is induced by two general types of chronic liver diseases, including hepatotoxic injury and cholestatic injury.^[^ ^138^ ^]^ Hepatotoxic injury is caused by infection with hepatitis virus B (HBV) or hepatitis virus C (HCV), alcoholic liver diseases, nonalcoholic fatty liver disease (NAFLD), and autoimmune liver diseases. Cholestatic injury is caused by primary sclerosing cholangitis, primary or secondary biliary cholangitis, and biliary atresia.^[^ ^139^ ^]^ Among these causes, HBV, HCV, and alcohol are the three most common aetiologies of DALYs from cirrhosis.^[^ ^140^ ^]^ Hepatic fibrosis is the leading cause of long‐term mortality,^[^ ^4^, ^141^, ^142^ ^]^ eventually leading to hepatic cirrhosis.^[^ ^143^ ^]^

#### 3.2.1 Origin, Heterogeneity and Role of Myofibroblasts in Hepatic Fibrosis

Myofibroblasts, which are the main source of ECM in hepatic fibrosis, are not present in healthy liver.^[^ ^144^ ^]^ Injured liver activates a large amount of myofibroblasts, which are potential therapeutic targets for hepatic fibrosis. Emerging evidence has revealed that myofibroblasts in fibrotic liver generally originate from liver‐resident fibroblasts, pericytes (e.g., hepatic stellate cells [HSCs]), portal fibroblasts and bone marrow‐derived fibrocytes and MSCs.^[^ ^64^ ^]^ Electron microscopy and other techniques have suggested that both HSCs and portal fibroblasts can transform into myofibroblasts.^[^ ^65^, ^66^, ^145^ ^]^ However, no evidence has shown that EMT or mesenchymal‐to‐epithelial transition (MET) contributes to hepatic fibrosis in mouse model.^[^ ^146^ ^]^ Different strain‐specific mouse models also present various susceptibilities to hepatic fibrosis (**Table** **3**). Therefore, the selection of appropriate mouse models is important for research on hepatic fibrosis.

**Table 3:** Disease‐specific and strain‐specific mouse models of hepatic fibrosis.

Model type	Strain type	Susceptibility to fibrosis	Reference
NAFLD	C57BL/6	Susceptible	[147]
	BALB/c	Resistant	
	PWK/PhJ	Susceptible	[148]
	CAST/EiJ	Resistant	
CCL4‐induced liver injury	BALB/c	Susceptible	[149]
	C57BL/6J	Resistant	
	BALB/cJ	Susceptible	[150]
	DBA/2J	Intermediate	
	C3H/HeJ	Intermediate	
	A/J	Resistant	
	AKR/J	Resistant	
	FVB/NJ	Resistant	
Carcinogenic diet‐induced liver injury	C57BL/6	Susceptible	[151]
	BALB/c	Resistant	
Schistosoma japonicum cercariae‐induced fibrosis	ICR	Susceptible	[152]
	C3H/He	Intermediate	
	CBA	Intermediate	
	NMRI	Intermediate	
	C57BL/6	Resistant	
	C57BL/Ks	Resistant	

CCL_4_, carbon tetrachloride; NAFLD, nonalcoholic fatty liver disease; NMRI, Naval Medical Research Institute, USA.

**Figure 4:**
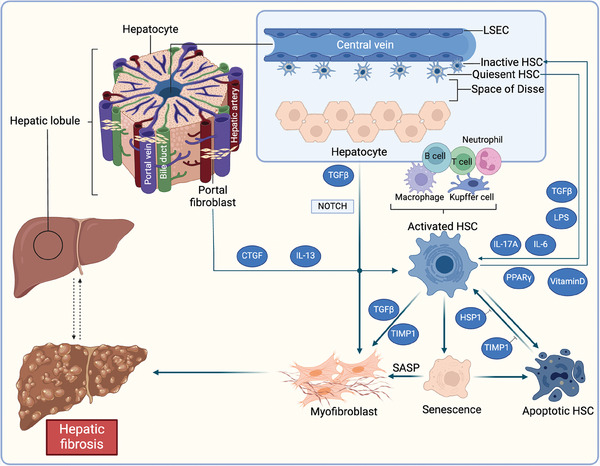
Mechanisms of hepatic fibrosis progression and regression. The portal vein, bile duct, and hepatic artery are shown in the structure of the hepatic lobule. Under normal conditions, portal fibroblasts reside in portal triads. The quiescent hepatic stellate cells (HSCs) are located in the space of Disse between sinusoidal endothelial cell and hepatocyte clusters. In response to fibrogenic stimulation, quiescent HSCs are activated by TGF‐β, LPS, IL‐6, and IL‐17A. Moreover, portal fibroblasts are also activated by connective tissue growth factor (CTGF) and IL‐13. Cellular senescence also promotes the conversion of senescent HSCs into myofibroblasts through the senescence‐associated secretory phenotype (SASP). Activated HSCs (with a myofibroblastic phenotype) differentiate into collagen type I‐producing cells to generate the extracellular matrix to promote hepatic fibrosis. Once stimulation ceases, approximately half of activated HSCs undergo apoptosis, which is inhibited by TIMP1 and HSP1. Nonapoptotic, activated HSCs undergo inactivation, which is facilitated by peroxisome proliferator‐activated receptor γ (PPARγ) and vitamin D. Immunocytes, such as lymphocytes, Kupffer cells, neutrophils, and macrophages also play bidirectional roles in both the progression and regression of hepatic fibrosis. LESC, sinusoidal endothelial cell; LPS, lipopolysaccharide. Created with Biorender.com.

HSCs, which constitute 10% of hepatic resident cells, have been shown to be the principal origins of ECM.^[^ ^153^, ^154^ ^]^ Under normal conditions, quiescent HSCs function as pericytes that reside in the space of Disse (**Figure** **4**). Persistent injury overactivated HSCs with characteristics of myofibroblasts, in which vitamin A, GFAP, and PPARγ are downregulated.^[^ ^64^ ^]^ A transcriptional roadmap for the activation of HSCs provided strong evidence that HSCs, rather than portal fibroblasts, suffer from dramatic transcriptomic alterations.^[^ ^155^ ^]^ Ingmar Mederacke et al. confirmed that 82–96% of hepatic myofibroblasts are transformed from HSCs in transgenic mouse models of toxic, cholestatic, and fatty liver disease.^[^ ^156^ ^]^ In addition, they also determined that HSCs are not progenitors of epithelial cells. Toxic liver injury primarily induces HSC activation, during which portal fibroblasts are also activated and contribute together to cholestatic hepatic fibrosis.^[^ ^157^, ^158^ ^]^ Moreover, Keiko Iwaisako and colleagues reported that activated liver‐resident HSCs and activated portal fibroblasts account for >95% of fibrogenic myofibroblasts in toxic and cholestatic hepatic fibrosis mouse models and that portal fibroblasts contribute >70% of myofibroblasts in cholestatic liver injury at the onset of injury (five days after bile duct ligation).^[^ ^157^ ^]^ During the progression of injury, the ratio of HSCs is greater than that of portal fibroblasts 14 and 20 days after bile duct ligation. Additionally, fibrocytes constitute approximately <4% of bone marrow‐derived myofibroblasts in hepatic fibrosis mouse models.^[^ ^159^ ^]^ The injury site recruits activated HSCs to produce ECM to form fibrous scars, during which several cytokines (e.g., TGF‐β) further trigger the activation of HSCs in an SMAD2‐dependent or SMAD3‐dependent manner.^[^ ^160^ ^]^ Moreover, IL‐13 and connective tissue growth factor (CTGF) promote the expression of COL1A1 in activated HSCs through a TGF‐β1‐dependent signaling pathway.^[^ ^161^ ^]^ HSCs then transdifferentiate into myofibroblastic cells with contractile, proinflammatory, and fibrogenic features. Recently, researchers found that deletion of hexokinase 2 inhibits HSC activation in hepatic fibrosis. Activated HSCs produce lactate, which determines the fate of HSCs through histone lactylation.^[^ ^162^ ^]^

Hepatocytes also respond to hepatic injury. Hepatocyte‐induced NOTCH activation was reported to be associated with NASH severity in a diet‐induced nonalcoholic steatohepatitis (NASH) mouse model.^[^ ^163^ ^]^ Hepatic fibrosis was alleviated in hepatocyte‐specific loss‐of‐function mouse models. Furthermore, deletion of receptor tyrosine kinase ephrin type B receptor 2 (RphB2), a downstream effector of the NOTCH signaling pathway in hepatocytes, can alleviate hepatic inflammation and fibrosis.^[^ ^164^ ^]^ In addition, specific deletion of the NOD‐like receptor 3 (NLRP3) inflammasome and downstream Gasdermin D attenuates hepatic fibrosis.^[^ ^165^ ^]^ Intriguingly, the interactions between cell components also contribute to hepatic fibrosis. For example, injured hepatocyte mitochondria release mitochondria‐derived damage‐associated molecular patterns (mito‐DAMPs) that directly activate HSCs, promoting hepatic fibrosis.^[^ ^166^ ^]^ Yuichi Tsuchiya et al. demonstrated increased expression of *Fgf18* in the livers of hepatic fibrosis mouse models.^[^ ^167^ ^]^ In this study, scRNA‐seq revealed that high expression of *Fgf18* in hepatocytes is along with elevated Lrat^+^ HSCs, which facilitate hepatic fibrosis. In addition, a positive feedback loop exists between TGF‐β and the DNA demethylase TET3 in both HSCs and hepatocytes.^[^ ^168^ ^]^ TGF‐β secreted by both cell types activates TET3 and triggers the expression of profibrotic genes. Therefore, hepatocytes alone may be not sufficient enough to lead to fibrosis.

In the healthy state, portal fibroblasts contribute to a small population of the hepatic fibroblast pool, which resides in the portal venules to maintain the physical integrity of the portal tract. Portal fibroblasts are now related to the pathogenesis of cholestatic hepatic injury.^[^ ^169^ ^]^ TGF‐β is essential for the activation of portal fibroblasts.^[^ ^138^ ^]^ Activated portal fibroblasts were reported to contribute to cholestatic fibrosis.^[^ ^170^ ^]^ This evidence suggests that portal fibroblasts are activated at the onset of cholestatic hepatic injury. Persistent hepatic injury transforms portal fibroblasts into myofibroblasts which are characterized by proliferation and production of the ECM.^[^ ^171^ ^]^ Unlike activated HSCs, activated portal fibroblasts do not respond to PDGFβ and NGF.^[^ ^157^ ^]^ IL‐25 activates portal fibroblasts to secrete IL‐13, which in turn promotes HSC activation by releasing CTGF. However, portal fibroblasts remarkably express ectonucleotidase 2 (NTPDase2), which is downregulated when these cells are transformed into myofibroblast‐like cells.^[^ ^172^, ^173^ ^]^ Thus, the origin of portal fibroblasts in hepatic fibrosis is still controversial.

Fibrocytes, which are derived from hematopoietic stem cells, mediate the tissue repair process and antigen presentation to naïve T cells.^[^ ^174^ ^]^ Fibrocytes can transform into myofibroblasts that contribute to fibrosis. In addition to type I collagen and fibronectin, fibrocytes also produce TGF‐β, which facilitates the accumulation of the ECM.^[^ ^175^ ^]^ The injury site recruits and transforms fibrocytes, which constitute 3–5% of the collagen‐expressing cells.^[^ ^176^, ^177^ ^]^

#### 3.2.2 Regression of Hepatic Fibrosis

Previous studies have shown that hepatic fibrosis is irreversible.^[^ ^178^ ^]^ However, researchers have reported that the liver can return to a normal state after removing the profibrotic aetiologies, which is also known as fibrosis regression.^[^ ^179^, ^180^ ^]^ For example, persistent inflammation, such as untreated hepatotoxic injury, promotes the progression of hepatic fibrosis, even liver failure and HCC.^[^ ^181^, ^182^ ^]^ Conversely, antiviral therapies targeting viral hepatitis and/or substantial lifestyle improvement in those with NASH can alleviate hepatic fibrosis.^[^ ^183^, ^184^, ^185^ ^]^ Additionally, abstinence effectively alleviates hepatic fibrosis in patients with alcoholic abuse.^[^ ^186^ ^]^ Weight loss or bariatric surgery attenuates insulin resistance and metabolic syndrome and reverses hepatic fibrosis.^[^ ^187^ ^]^

Hepatocytes, activated HSCs, endothelial cells, and immunocytes are involved in the regression of hepatic fibrosis.^[^ ^74^ ^]^ One possible mechanism is that activated HSCs spontaneously initiate apoptosis or escape apoptosis and revert to an inactive phenotype.^[^ ^188^ ^]^ Depletion of activated HSCs leads to regression of hepatic fibrosis in several experimental models.^[^ ^189^ ^]^ Approximately half of activated HSCs or myofibroblasts die after the cessation of CCL_4_‐induced hepatic fibrosis,^[^ ^190^, ^191^ ^]^ during which senescent HSCs inhibit BCL2 expression and ECM production. Additionally, the number of senescent‐activated HSCs increases during the regression of hepatic fibrosis. Cellular senescence is characterized by irreversible cell cycle arrest that remains metabolically active, and is triggered by DNA damage, oncogene activation, or other stress‐mediated signals.^[^ ^192^, ^193^ ^]^ The role of activated HSC senescence in fibrosis is still controversial. Cellular senescence promotes fibrosis progression through the senescence‐associated secretory phenotype (SASP). However, senescent‐activated HSCs exhibit gene expression parallel to cell‐cycle exit, decreased secretion of ECM, increased production of ECM‐degrading enzymes, and increased immune surveillance, restricting hepatic fibrosis. The opposite phenomenon in which senescent HSCs play a role in hepatic fibrosis is incomplete characterization of the senescent phenotype and cell subpopulation. Therefore, clarification of HSC subtypes facilitates the investigation of therapeutic targets for the regression of hepatic fibrosis.

Immunocytes are also critical components that contribute to the resolution of hepatic fibrosis. Resident macrophages in the liver, also known as Kupffer cells, express chemokines at the early stage of liver injury. Kupffer cells recover during the regression of hepatic fibrosis.^[^ ^194^ ^]^ However, the origin and mechanism of the repopulation of Kupffer cells remain unclear. Emerging evidence shows that infiltrating macrophages, which are recruited through CCR2/CCL2 signaling, play dual roles in promoting and resolving fibrosis.^[^ ^45^, ^194^ ^]^ On the one hand, macrophages stay close to HSCs at fibrotic septa to facilitate hepatic fibrosis via the generation of profibrotic factors.^[^ ^74^, ^195^ ^]^ Disruption of the Mucosal‐Associated Invariant T (MAIT)‐monocyte/macrophage interaction leads to regression of hepatic fibrosis by increasing restorative Ly6CIo and promoting an autophagic phenotype in macrophages.^[^ ^196^ ^]^ On the other hand, macrophages also promote the apoptosis of myofibroblasts through the expression of MMP9 and TRAIL,^[^ ^194^ ^]^ which induce the production of ECM degradation‐associated MMPs (e.g., MMP12 and MMP13) to resolve hepatic fibrosis.^[^ ^197^, ^198^ ^]^ Additionally, researchers have demonstrated that anti‐inflammatory senescent macrophages determine the degree of hepatic fibrosis regression. Once macrophage senescence is depleted, the regression of hepatic fibrosis is abolished.^[^ ^199^ ^]^ Recent studies have focused on the effects of engineering macrophages with a chimeric antigen receptor (CAR) on hepatic fibrosis. They reported that the adoptive transfer of CAR‐Macrophages results in a significant reduction in hepatic fibrosis through regulation of the immune microenvironment in murine models.^[^ ^200^ ^]^

In addition, the activation of liver‐associated natural killer (NK) cells may decrease hepatic fibrosis by killing activated HSCs. Activated HSCs, not hepatocytes, express the death receptor TRAIL, which binds to NK cells, thereby inducing the apoptosis of HSCs.^[^ ^201^ ^]^ Other immune cells, such as T cells, also play major roles in determining hepatic fibrosis. In parasitic infection models, T helper 1 (Th1) cell responses trigger the regression of fibrosis, whereas the induction of Th2 responses promotes fibrosis.^[^ ^202^ ^]^ Natural Tregs also contribute to the alleviation of fibrosis during parasitic infection via the production of IL‐10.^[^ ^74^ ^]^ The current understanding of the principal components and interactions between HSCs and immunocytes contributes to the investigation of therapeutic targets for the regression of hepatic fibrosis.

### 3.3 Pulmonary Fibrosis

Idiopathic pulmonary fibrosis (IPF), the most severe outcome of idiopathic interstitial pneumonia, is a chronic, progressive, and age‐related disease.^[^ ^203^ ^]^ IPF is characterized by abnormal accumulation of ECM and stromal cells that destroy the alveolar architecture, which results in worsening respiratory symptoms, leading to respiratory failure.^[^ ^79^ ^]^ The prevalence of IPF (per 10,000 of the population) in Asia‐Pacific countries ranges from 0.57‐4.51, 0.33‐2.51 in Europe, and 2.40–2.98 in North America, with increasing mortality and morbidity.^[^ ^204^, ^205^ ^]^ The mean age of IPF patients is 65–70 years.^[^ ^206^ ^]^ The aetiologies of IPF are varied and unclarified, possibly including allergens, toxic chemicals, radiation, and environmental exposures.^[^ ^207^ ^]^ These potential factors have led to an increased prevalence of IPF over the last two decades.^[^ ^208^ ^]^

#### 3.3.1 Origin and Role of Myofibroblasts in Pulmonary Fibrosis

Activated myofibroblasts are the main source of ECM in pulmonary fibrosis.^[^ ^209^ ^]^ However, the origins of myofibroblasts in IPF are still poorly understood. The primary sources of myofibroblasts include resident cells (fibroblasts, pericytes, and mesenchymal progenitor cells), circulating bone marrow‐derived fibrocytes, epithelial cells, mesothelial cells, and endothelial cells that can transform into myofibroblasts.^[^ ^77^, ^80^ ^]^ Morphologically, α‐SMA is an important marker of myofibroblasts characterized by increased contractility, reduced motility, and proliferation.^[^ ^210^ ^]^ Importantly, the cytoskeletal composition is different among myofibroblasts due to their localization. Therefore, fibrotic lung resident myofibroblasts express vimentin and α‐SMA without desmin, except for those in peripheral and subpleural regions of the lung.^[^ ^211^ ^]^

Three common mouse models of pulmonary fibrosis are bleomycin‐induced, radiation‐induced, and silicosis‐induced pulmonary fibrosis. However, the pathophysiological process of fibrosis differs among various strain‐specific mouse models (**Table** **4**). In a mouse model susceptible to profibrotic stimulation, lung tissue damage stimulates the activation of myofibroblasts to secrete ECM, a reservoir of growth factors and related signaling, in the lung interstitium.^[^ ^212^ ^]^ IPF also promotes the translation of ECM proteins, including COL1A1, COL1A2, COL3A1, MMP2, MMP3, and TIMP2.^[^ ^213^ ^]^ Thus, a positive feedback loop between fibroblasts and a stiff ECM is formed, resulting in severe fibrosis. Excessive deposition of ECM changes both the biochemical microenvironment and the mechanical properties of fibrotic lung tissue.^[^ ^214^ ^]^ High mechanical properties, stimulation by TGF‐β, and the presence of specific matrix proteins are three major driving forces of the differentiation of fibroblasts to myofibroblasts.^[^ ^215^ ^]^ Increased mechanical properties, such as ECM stiffness, as measured by the Young's modulus, of the lung matrix promote differentiation into myofibroblasts.^[^ ^216^, ^217^ ^]^ In general, normal lung tissue is soft and elastic with a Young's modulus of ≈1 kPa to allow air exchange. However, the rigidity of lung tissue in IPF patients sharply increases due to the accumulation of myofibroblasts with an elevated modulus of 30–50 kPa.^[^ ^216^ ^]^ Furthermore, mechanosensitive α‐integrin on myofibroblasts plays a critical role in promoting fibrosis via the activation of TGF‐β.^[^ ^218^, ^219^ ^]^ A stiff matrix promotes pulmonary fibrosis by activating β1 integrin and focal adhesion kinase (FAK) in fibroblasts.^[^ ^220^, ^221^ ^]^ This evidence suggests that the integrin signaling pathway functions in integrating several signals to regulate myofibroblast transformation.^[^ ^222^ ^]^

**Table 4:** Disease‐specific and strain‐specific mouse models of pulmonary fibrosis.

Model type	Strain type	Susceptibility to fibrosis	Reference
Bleomycin‐induced pulmonary fibrosis	C57BL/6J	Susceptible	[223, 224]
	C3Hf/Kam	Resistant	[223]
	C57BL/6	Susceptible	[225, 226, 227]
	DBA/2	Intermediate	[225, 227]
	DBA/2J	Intermediate	[225]
	Swiss	Intermediate	[225]
	BALB/c	Resistant	[225, 227]
	NMRI	Susceptible	[226]
	A/J	Resistant	[224]
Radiation‐induced pulmonary fibrosis	C57BL/6J	Susceptible	[228]
	C3Hf/Kam	Resistant	
	C57BL/6NHsd	Susceptible	[229]
	C3H/HeNHsd	Resistant	
Silicosis‐induced pulmonary fibrosis	C3H/HeN	Susceptible	[230]
	New Zealand Black mice	Susceptible	
	BALB/c	Resistant	
	MRL/MpJ	Resistant	

#### 3.3.2 The Role of Alveolar Epithelial Cells in Pulmonary Fibrosis

**Figure 5:**
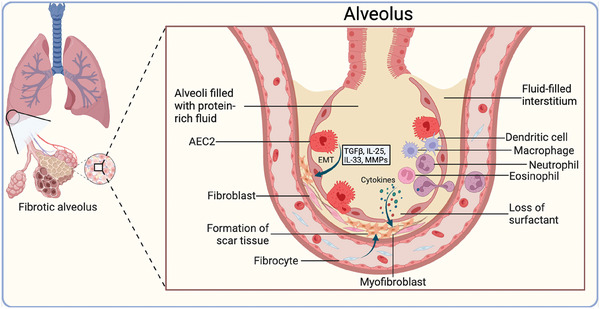
The mechanisms of pulmonary fibrosis and the origin of myofibroblasts. Pulmonary injury induces the accumulation of protein‐rich fluid in both the alveoli and interstitium. In addition to resident fibroblasts, recruited fibrocytes through blood flow, alveolar epithelial type II cells (AEC2s) and immunocytes are also sources of myofibroblasts in the early stage of pulmonary fibrosis. The recruited immunocytes, including macrophages, neutrophils, eosinophils, and dendritic cells, secrete chemokines and cytokines (such as TGF‐β, IL‐4, IL‐13, IL‐17, IL‐22, and IFNγ) that facilitate inflammation. These fibrogenic factors promote apoptosis, abnormal proliferation, and ineffective migration of alveolar epithelial cells. However, these factors trigger myofibroblast transformation, resistance to apoptosis, and extracellular matrix deposition. Upon fibrogenic stimulation, macrophages are activated to change their phenotype to a myofibroblastic phenotype. The cytokines secreted by inflammatory cells also modulate the transformation of AEC2s to myofibroblasts. The excessive accumulation of ECM in the alveoli destroys the structure and function of the alveoli, thus leading to dysregulation of gas exchange and pulmonary fibrosis. EMT, epithelial‐to‐mesenchymal transformation. Created with Biorender.com.

The primary functions of epithelial cells in fibrosis are cell proliferation, apoptosis, senescence, aging, and EMT^[^ ^213^ ^]^ (**Figure** **5**). For example, alveolar epithelial type II cells (AEC2s) function in secretion and regeneration in the alveolus.^[^ ^231^ ^]^ However, damage to AEC2s results in a profibrotic phenotype that leads to pulmonary fibrosis. In addition, multiple studies have revealed that the apoptosis of AEC2s in the process of fibrogenesis might be one of the foremost events in response to injury to the epithelium.^[^ ^232^ ^]^ Interestingly, AEC aging has also been found to be a key driver of fibrosis. Cluster of differentiation 38 (CD38), a cardinal nicotinamide adenine dinucleotide (NAD) hydrolase, is a marker of senescent cells and is highly expressed in the AEC2s of patients with pulmonary fibrosis, which serves as a potential therapeutic target.^[^ ^233^ ^]^ Researchers have discovered that CD38 AECs are negatively associated with the pulmonary functions of patients.^[^ ^234^ ^]^ Similarly, increased expression of CD38 in the AECs of fibrotic lungs was detected in young mice and further strengthened in aged mice. CD38 antigen receptor membrane‐modified MSC‐derived extracellular vesicle (CD38‐ARM‐MSC‐EV) associated technology is a promising agent with high clinical potential against pulmonary fibrosis.^[^ ^233^ ^]^

Pulmonary epithelial cell injury activates AECs that function similarly to myofibroblasts. A previous study revealed that AEC‐derived type I collagen significantly activated the fibroblast collagen receptor discoidin domain receptor 2 and increased the secretion of ECM, leading to fibrogenesis.^[^ ^235^ ^]^ The secretion of mediators (e.g., TGF‐β, PDGF, TNF, MMPs, osteopontin, and chemokines) by injured AECs in fibrotic lungs is the main factor contributing to ECM remodeling.^[^ ^81^, ^236^ ^]^ These mediators directly activate myofibroblasts, or promote AEC transformation to produce ECM. For example, TGF‐β‐dependent EMT in human AEC2s was found to involve actin cytoskeleton remodeling, differentiation processes, and collagen secretion.^[^ ^237^ ^]^ Additionally, AECs also secrete coagulation‐associated factors, including tissue factor, factor VIIa/X, and plasminogen activator inhibitor I, which are profibrotic factors.

Emerging evidence has demonstrated that AEC senescence occurs in IPF.^[^ ^238^, ^239^, ^240^ ^]^ Aberrant telomere shortening in fibrotic lungs causes cellular senescence in AECs.^[^ ^241^ ^]^ A Mendelian randomization inference of the length of telomeres (indicating cell senescence) causality in IPF from the UK Biobank revealed that shortened telomeres led to IPF.^[^ ^242^ ^]^ However, the mechanisms regarding the aging‐related susceptibility to pulmonary fibrosis are largely unknown.^[^ ^243^ ^]^ The accelerated senescence of AECs is one of the major mechanisms inducing abnormal activation of AECs in response to injury, which promotes the deposition of ECM and destruction of the lung architecture.^[^ ^244^ ^]^ Senescence is related to diverse secretomes, which provide various stimuli and cellular microenvironments. A two‐stage senescent model is proposed for this process.^[^ ^245^ ^]^ In the first stage, epithelial cells and fibroblasts undergo a prosenescent and profibrotic NOTCH1‐induced TGF‐β‐rich secretome that inhibits the secretion of proinflammatory cytokines and MMPs. In the second stage, activation of the transcription factors of the C/EBPβ family triggers a proinflammatory, matrix‐degrading, and senescence‐clearing mechanism, leading to ECM degradation. In addition, researchers have reported that elevated phosphatase tensin homolog (PTEN)/NF‐κB is associated with the senescence of AECs in human lung tissue.^[^ ^246^, ^247^ ^]^ Senescence markers and the SASP are augmented in AECs treated with bleomycin in vitro. Moreover, AEC2s in fibrotic lungs exhibit a strongly senescent phenotype such as mitochondrial malformation and dysfunction.^[^ ^248^ ^]^ Recent studies have demonstrated that mitochondrial dysfunction and metabolic reprogramming occur in pulmonary fibrosis.^[^ ^249^ ^]^ Mitochondria produce reactive oxygen species (ROS) and ATP to support cellular metabolism. During AEC senescence, morphological changes in mitochondria (e.g., rounded appearance, cristae, and inner membrane absence), decreased mitochondrial DNA copy count and reduced biogenesis of mitochondria controlled by peroxisome proliferator‐activated receptor γ coactivator 1α (PGC‐1α) affect ATP production.^[^ ^250^, ^251^, ^252^ ^]^ Currently, several studies have focused on investigating therapeutic approaches that target cellular senescence in pulmonary fibrosis. The RNA‐binding protein YTHDC1, which is expressed mainly in AEC2s, alleviates AEC senescence by promoting the interaction between TopBP1 and MRE11, thereby facilitating DNA repair.^[^ ^253^ ^]^ Leucine‐rich repeat kinase 2 (LRRK2) in AEC2s sharply decreases in the lungs of bleomycin‐treated mice, and its deficiency leads to accelerated cellular senescence.^[^ ^254^ ^]^ Hence, LRRK2 plays a critical role in preventing pulmonary fibrosis.

#### 3.3.3 Inflammatory Immunopathogenesis of SARS‐CoV‐2 in Pulmonary Fibrosis

In addition to fibroblasts and epithelial cells, immunocytes also function in pulmonary fibrosis. The coronavirus disease 2019 (COVID‐19) pandemic, which is caused by severe acute respiratory syndrome coronavirus 2 (SARS‐CoV‐2), has caused injury to AEC2s and recruited immunocytes, resulting in a cytokine storm and leading to lung tissue remodeling.^[^ ^221^ ^]^ Histopathological alterations in the lungs suppress gas exchange, resulting in hypoxemia.^[^ ^255^ ^]^ Pulmonary fibrosis occurs not only during SARS‐CoV‐2 infection, but also in the recovery stage after discharge.^[^ ^256^ ^]^ Another study revealed that approximately one‐third of 397 patients recovered from pulmonary fibrosis 120 days after onset.^[^ ^257^ ^]^ Although the overproduction of proinflammatory factors is currently considered the main cause of SARS‐CoV‐2‐induced pulmonary fibrosis, the specific molecular mechanisms are still largely unknown. The role and application of available antifibrotic therapies in patients with SARS‐CoV‐2‐induced IPF are poorly understood.^[^ ^258^ ^]^

**Figure 6:**
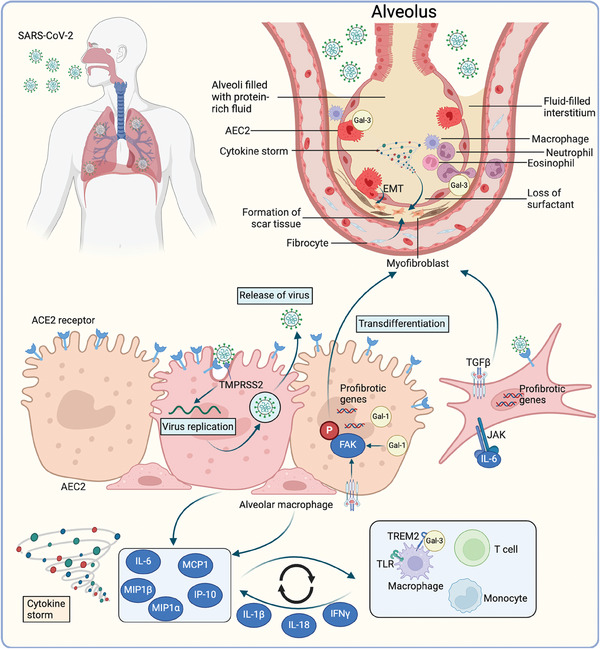
Molecular mechanisms of SARS‐CoV‐2‐induced pulmonary fibrosis. When SARS‐CoV‐2 is inhaled into the respiratory system of the human body, the virus enters the host cell with the assistance of the angiotensin‐converting enzyme 2 (ACE2) receptor and TMPRSS2. The active replication and release of the virus through exocytosis lead to a proinflammatory response and pyroptosis. These signals and patterns are recognized by adjacent epithelial cells, endothelial cells, and alveolar macrophages, facilitating the production of cytokines and chemokines (such as IL‐6, MCP1, IP‐10, MIP1α, and MIP1β). These factors recruit multiple immunocytes, including macrophages, T cells, and monocytes, and promote more severe inflammation. Monocyte/macrophage‐derived IL‐1β and epithelial cell‐derived IL‐6 contribute to the proinflammatory feedback loop and cause overproduction of cytokines, resulting in a cytokine storm. Galectin‐3 (Gal‐3) also binds to Toll‐like receptor 4 (TLR4) to facilitate pulmonary fibrosis. Gal‐1 promotes transdifferentiation from alveolar epithelial type II cells (AEC2) to myofibroblasts via the FAK signaling pathway. Alveolar fibroblasts can also be activated by viral response‐induced TGF‐β signaling, JAK/STAT signaling, and direct interactions with AEC2s. Created with Biorender.com.

SARS‐CoV‐2 is transmitted mainly through respiratory droplets. Upon infection, SARS‐CoV‐2 enters the cells expressing the surface receptors angiotensin‐converting enzyme 2 (ACE2) and TMPRSS2, leading to replication and release of the virus^[^ ^259^ ^]^ (**Figure** **6**). The damage‐associated molecular patterns in infected cells, including ATP, nucleic acids, and ASC oligomers, are recognized by adjacent epithelial cells, endothelial cells, and alveolar macrophages. These activated cells produce proinflammatory cytokines and chemokines, including IL‐6, IP‐10, monocyte chemotactic protein 1 (MCP1), interferon γ (IFNγ), and macrophage inflammatory protein 1α (MIP1α).^[^ ^260^, ^261^, ^262^ ^]^ These proinflammatory factors recruit monocytes, T cells, and macrophages to the infection site, causing a positive feedback loop in the overproduction of cytokines that results in a cytokine storm. On the one hand, these proinflammatory mediators and angiotensin II induce the synthesis of MMPs and lead to pathological remodeling of the ECM in the lung.^[^ ^263^ ^]^ On the other hand, cytokines promote the proliferation and migration of fibroblasts, thereby leading to ECM deposition.

Cellular senescence is another modulator of SARS‐CoV‐2‐induced hyperinflammation. Infection with SARS‐CoV‐2 exacerbates SASP, which involves proinflammatory, ECM‐degrading, complement‐activating and procoagulatory factors synthesized by senescent cells.^[^ ^264^ ^]^ These factors can lead to cytokine storm and tissue‐destructive immunocyte infiltration, which can inversely accelerate cellular senescence and result in pulmonary fibrosis.

Recent studies have identified multiple molecular mechanisms with potential to be therapeutic targets. Galectin (Gal) generally binds to sugar residues that function in cell interactions, adhesion, and transmembrane signaling. Recent studies revealed that Gal‐1 is involved in the progression of IPF. In AECs, FAK1 is activated by Gal‐1 and mediates the transdifferentiation of myofibroblasts, resulting in pulmonary fibrosis.^[^ ^265^ ^]^ Gal‐3, which is generally expressed in endothelial cells, fibroblasts, and resident alveolar macrophages, is the most studied galectin in pulmonary fibrosis caused by SARS‐CoV‐2. Gal‐3 not only activates the inflammatory response and cytokine storm in the lungs but also triggers TGF‐β signaling and leads to EMT, ECM accumulation, and epithelial cell apoptosis.^[^ ^266^, ^267^ ^]^ In addition, Gal‐3 can also lead to pulmonary fibrosis through binding to Toll‐like receptor 4 (TLR4).^[^ ^268^ ^]^ TLRs function mainly in the recognition of virus particles and activation of the innate immune system. The activation of TLRs promotes the production of proinflammatory cytokines, such as IL‐1, IL‐6, and TNFα.^[^ ^269^ ^]^

Compared with other viral and bacterial causes of pneumonia, monocyte/macrophage‐derived IL‐1β and epithelial cell‐derived IL‐6 are specific characteristics of SARS‐CoV‐2‐induced pneumonia.^[^ ^270^ ^]^ Human airway epithelia, which is infected by SARS‐CoV‐2, generates signals (i.e., genomic and mitochondrial DNA) to promote the release of IL‐1β by leukocytes. The released IL‐1β stimulates the airway epithelia to secrete IL‐6.^[^ ^271^ ^]^ The infection and replication of SARS‐CoV‐2 in human lung macrophages is another critical driver of pneumonia. Human macrophages promote inflammasomes in response to infection triggered by CD16 and ACE2 receptors, resulting in the release of IL‐1 and IL‐18 to induce inflammation.^[^ ^272^ ^]^ CD147 is an adhesion molecule that also functions in mediating inflammation and the immune response. Previous studies revealed that CD147‐spike protein is a universal route for SARS‐CoV‐2 infection in host cells.^[^ ^273^, ^274^ ^]^ A recent study revealed that CD147 is a major modulator of SARS‐CoV‐2‐induced fibroblast activation.^[^ ^275^ ^]^ Exploration of the underlying mechanisms is of great importance for the early intervention in SARS‐CoV‐2‐induced pulmonary fibrosis.

### 3.4 Cardiac Fibrosis

Cardiac fibrosis, which is characterized by extensive ECM deposition in the cardiac interstitium, occurs in multiple heart diseases, such as myocardial infarction (MI), cardiac hypertrophy, diabetic cardiomyopathy, and dilated cardiomyopathy.^[^ ^276^, ^277^, ^278^ ^]^ Heart failure (HF) is a complex clinical syndrome with a prevalence of 23 million individuals globally and high morbidity and mortality rates.^[^ ^279^, ^280^ ^]^ Cardiac fibrosis is regarded as a requisite that exists in HF.^[^ ^281^ ^]^ Importantly, despite the negative association between cardiac fibrosis and prognosis, fibrosis is not the primary factor of cardiac dysfunction.^[^ ^282^ ^]^ The regeneration capacity of adult mammalian hearts is negligible; thus, the formation of a fibrous scar is a common process for tissue repair. Microscopically, cardiac fibrosis manifests as two patterns of ECM accumulation, including replacement or reparative fibrosis, reactive fibrosis, and interstitial fibrosis.^[^ ^283^, ^284^ ^]^ Replacement or reparative cardiac fibrosis indicates cardiomyocyte loss and replacement by ECM components, destroying the architecture of muscle bundles without damaging tissue integrity, such as during MI.^[^ ^285^, ^286^ ^]^ An immediate infarction leads to the death of cardiomyocytes and the formation of a collagen‐based scar. Reactive fibrosis refers to prolonged profibrotic stimulation with expansion of interstitial and perivascular spaces in the absence of cardiomyocyte loss, which is caused by pressure or volume overload, and diabetes mellitus.^[^ ^282^, ^287^ ^]^ Reactive fibrosis is generally induced by myocardial stress or chronic inflammation that causes ventricular stiffening, dysfunction, and HF.

#### 3.4.1 Origin and Role of Myofibroblasts in Cardiac Fibrosis

**Figure 7:**
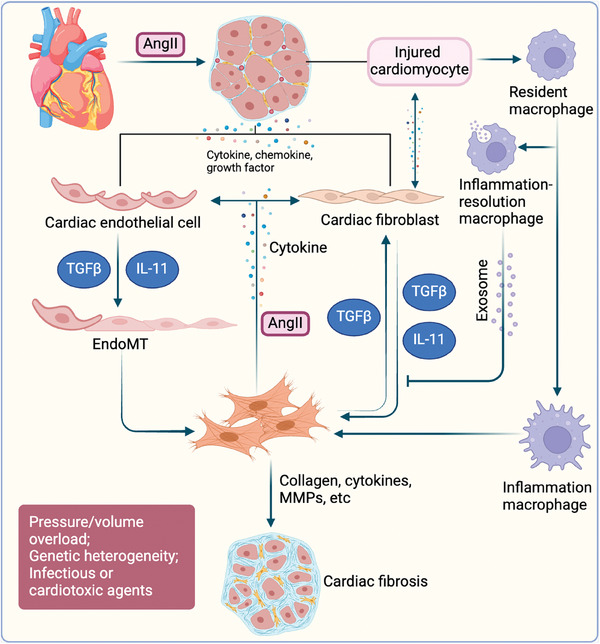
Mechanisms of cardiac fibrosis and the origin of myofibroblasts. Cardiac injury facilitates the secretion of fibrogenic factors, such as cytokines, chemokines, and growth factors. These fibrogenic factors promote transdifferentiation from cardiac fibroblasts to myofibroblasts and endothelial‐to‐mesenchymal transformation (EndoMT), leading to ECM accumulation in the cardiac interstitium. Injured cardiomyocytes activate resident macrophages to accelerate cardiac fibrosis. However, the proresolution subtypes inhibit myofibroblast differentiation through a paracrine approach. Ang II, Angiotensin II. Created with Biorender.com.

Cardiac fibroblasts, which originate from the proepicardium, endothelial cells, and neural crest cells, are the major stromal cells involved in maintaining the ECM structure and homeostasis.^[^ ^288^, ^289^, ^290^ ^]^ Cardiac fibroblasts are located mainly in the adventitia, interstitium, atrium, annulus, and valves, and have different functions and gene expression profiles.^[^ ^291^ ^]^Cardiac fibroblasts are responsible for maintaining cardiac function, mechanical and electrical transduction in the heart.^[^ ^292^ ^]^ Cardiac fibroblasts are also the main source of MMPs and four known TIMPs.^[^ ^293^, ^294^ ^]^ In addition, cardiac fibroblasts respond to injuries to the heart. When injury occurs, cardiac fibroblasts secrete a variety of proinflammatory cytokines, chemokines, and growth factors, such as IL‐1β, IL‐6, IL‐9, IL‐10, IL‐11, IL‐12, IFNγ, TNFα, CCL5, MCP‐1, and TLR4^[^ ^86^, ^295^, ^296^, ^297^, ^298^, ^299^ ^]^(**Figure** **7**). Fibroblasts are activated to secrete more ECM components to maintain the integrity of the heart. However, severe injury leads to excess accumulation of ECM, which reduces compliance of the ventricle and accelerates HF.

In multiple pathological conditions of the heart, cardiac fibroblasts are activated in the mesenchymal region for tissue repair. Resident fibroblasts exhibit heterogeneity in the health and disease status of the heart. Using single‐cell and single‐nucleus transcriptomes, researchers have demonstrated that seven populations of cardiac fibroblasts express *DCN*, *GSN*, and *PDGFRα* in the fibroblast compartment.^[^ ^300^ ^]^ Additionally, distinct chamber‐specific ECM‐producing cardiac fibroblasts with properties expressing different collagen isoforms and other ECM‐associated genes have been identified. This largely overcomes the lack of specific markers for identifying fibroblasts and fibroblastic cells.

Cardiac myofibroblasts are rarely observed in normal hearts, whereas myofibroblasts are largely transdifferentiated from fibroblasts and endothelial cells in response to injury.^[^ ^87^ ^]^ On the one hand, activated myofibroblasts assist in the formation of scars to prevent the ventricle from rupturing. On the other hand, excessive remodeling of the heart ultimately leads to fibrosis. Current opinions demonstrate that tissue‐resident fibroblasts are the primary source of cardiac myofibroblasts in the injury‐induced cardiac remodeling process.^[^ ^301^, ^302^ ^]^ Epithelial cells in the epicardium transdifferentiate into mesenchymal cells through EMT and acquire properties in migration, thus invading the region among cardiac myocytes to develop into another source of cardiac fibroblasts.^[^ ^92^, ^303^ ^]^ Endothelial cells are stable in healthy hearts. Endocardial cells can transdifferentiate into fibroblasts through EndoMT in the injured heart, which was first identified in a stress‐induced cardiac fibrosis mouse model.^[^ ^42^, ^304^ ^]^ In the model, endothelial cell‐derived fibroblasts accounted for 27–35% of all fibroblasts in the injured heart. Approximately 70% of myofibroblasts are transdifferentiated from EndoMT in the heart with pressure overload.^[^ ^42^, ^301^ ^]^ One important trigger of myofibroblast transdifferentiation is alterations in the biomechanical microenvironment, such as pressure or volume overload, genetic heterogeneity, and the use of infectious or cardiotoxic agents. The loss of tissue integrity disturbs the crosslinked ECM framework, allowing mechanical cues to promote the transition.^[^ ^305^ ^]^ The secretory phenotype of myofibroblasts includes type 1 collagen, TGF‐β, and α‐SMA, which promote the transdifferentiation of other cells to myofibroblasts.^[^ ^87^ ^]^ Angiotensin II, which can also be secreted by cardiac myofibroblasts, facilitates the production of the ECM through TGF‐β or type I angiotensin receptor (AT1R).^[^ ^306^, ^307^ ^]^ In addition, systemic extracardiac profibrotic alterations, such as diabetes and CKD, facilitate cardiac fibrosis. These contributors are regulated by various mechanisms, including the renin‐angiotensin‐aldosterone system, cytokines, chemokines, metabolic and mechanosensitive signaling, and growth factors.^[^ ^308^ ^]^ Interestingly, myofibroblasts themselves secrete various cytokines that function in the communication between cardiac inflammation and fibrosis.^[^ ^309^ ^]^ Additionally, the mediators that function in the activation of cardiac fibroblasts have different effects on the secretome of myofibroblasts.^[^ ^310^ ^]^ Overall, cardiac fibroblasts and myofibroblasts are the principal cell types that contribute to heart repair and fibrosis.

#### 3.4.2 Role of Other Cells in Cardiac Fibrosis

Previous studies have demonstrated that resident fibroblasts and myofibroblasts are the principal cell types involved in cardiac fibrosis, with limited contributions from other cell types.^[^ ^301^ ^]^ Researchers have found that OTU domain‐containing protein 1 (OTUD1) in cardiomyocytes promotes pathological cardiac fibrosis via signal transducer and activator of transcription 3 (STAT3) deubiquitination.^[^ ^311^ ^]^ Deletion of OTUD1 in mice prevents cardiac dysfunction, hypertrophy, and fibrosis induced by angiotensin II. However, not all mouse models of cardiac fibrosis, such as C57BL/6, are susceptible to angiotensin II. Different strain‐specific mouse models have various susceptibilities to stimulation (**Table** **5**).

**Table 5:** Disease‐specific and strain‐specific mouse models of cardiac fibrosis.

Model type	Strain type	Susceptibility to fibrosis	Reference
Myocardial injury	MRL/MpJ	Susceptible	[312]
	MRL/MpJ	Resistant	[313]
Angiotensin II‐induced cardiac fibrosis	C57BL/6	Resistant	[130]
Isoproterenol‐induced cardiac fibrosis	A/J	Susceptible	[314]
	C57BL/6	Resistant	

In addition, the crosstalk between cardiomyocytes and fibroblasts contributes to cardiac fibrosis.^[^ ^315^ ^]^ For example, damaged cardiomyocytes can also release alarmins, such as TGF‐β, to trigger fibroblast activation.^[^ ^89^, ^316^ ^]^ Vascular endothelial cells contribute to cardiac fibrosis through multiple mechanisms. The recruitment of leukocytes requires endothelial activation via increased expression of adhesion molecules and secretion of TGF‐β and endothelin‐1.^[^ ^317^, ^318^, ^319^ ^]^ Endothelial‐specific activation of the transcription factor forkhead box O4 (FoxO4) following MI has been reported to facilitate the infiltration of neutrophils into infarcted areas.^[^ ^320^ ^]^ Intriguingly, cardiac myofibroblasts can recruit leukocytes through granulocyte‐macrophage colony‐stimulating factor (GM‐CSF), chemokines, cytokines and other molecules.^[^ ^321^, ^322^ ^]^ Additionally, cardiac resident macrophages have been demonstrated to prevent fibrosis and stimulate angiogenesis.^[^ ^323^ ^]^ Depletion of resident macrophages worsen cardiac fibrosis. However, the polarization of macrophages from resident CCR2^+^ macrophages/monocytes to M1‐like macrophages results in the production of proinflammatory and profibrotic factors that induce cardiac fibrosis.^[^ ^95^ ^]^

## 4 Critical Signaling Pathways in Fibrosis

With the development of in‐depth research on the mechanisms of fibrosis, many signaling cascades involved in modulating myofibroblast activation, EMT, EndoMT, ECM accumulation, immunocyte infiltration, inflammation, and metabolism have been identified. In this section, we focus mainly on the clarified and canonical signaling pathways involved in fibrosis.

### 4.1 TGF‐β Signaling Pathway

TGF‐β is a key profibrotic cytokine and major mediator of fibrosis. The pro‐TGF‐β monomer is initially synthesized in the ribosome and folds in the ER.^[^ ^324^ ^]^ The latent pro‐TGF‐β complex in the ECM is related to a single peptide in the large N‐terminal portion termed latency‐associated peptide (LAP).^[^ ^325^ ^]^ LAP binds to latent TGF‐β‐binding proteins (LTBPs), which are key to latent TGF‐β localization and activation,^[^ ^326^ ^]^ covalently through two disulfide bonds.^[^ ^325^ ^]^ TGF‐β1, TGF‐β2, and TGF‐β3 are three isoforms of TGF‐β that are expressed only in mammals.^[^ ^327^ ^]^ TGF‐β can be activated by several proteases, integrins, and other factors in multiple cell types and tissues.^[^ ^328^ ^]^ Activated TGF‐β1, which is mainly secreted and stored in the ECM, interacts with the TGF‐βR1/2 complex to regulate biophysical functions through canonical (SMAD) and/or noncanonical (non‐SMAD) signaling pathways.

#### 4.1.1 Canonical Signaling Pathway

**Figure 8:**
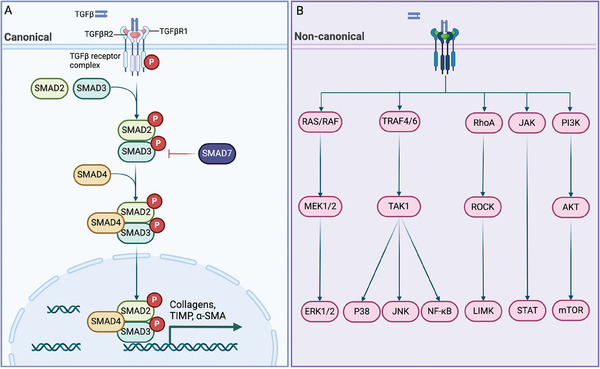
Overview of the canonical and noncanonical TGF‐β signaling pathways. A) In canonical SMAD‐dependent signaling, mature TGF‐β phosphorylates TGF‐βR2, which triggers the interaction between TGF‐βR1 and phosphorylates receptor‐SMAD proteins. Co‐SMAD (SMAD4) and phosphorylated R‐SMAD (SMAD2/3) translocates into the nucleus to modulate the expression of downstream genes, such as collagens, TIMPs, and α‐SMA. B) Mature TGF‐β also functions via noncanonical signaling pathways, such as the RAS/RAF, TRAF4/6, RhoA/ROCK, JAK/STAT, and PI3K/AKT pathways. Created with Biorender.com.

SMAD proteins, which are canonical effectors of TGF‐β/ TGF‐βR, are recognized by TGF‐βR2, which recruits and phosphorylates TGF‐βR1 (**Figure** **8**A). Activated TGF‐βR1 phosphorylates receptor‐SMAD (R‐SMAD) and induces the interaction between R‐SMAD and Co‐SMAD/SMAD4, which relocates into the nucleus to regulate target genes as transcription factors.^[^ ^329^ ^]^ In addition, downstream SMAD2/SMAD3 are considered critical effectors of TGF‐β signaling in fibrosis and tumorigenesis. Our previous study demonstrated that pathological mechanical stimulation can induce bladder fibrosis via SMAD3 signaling.^[^ ^6^ ^]^ However, SMAD6/SMAD7 act as negative regulators of TGF‐β signaling.^[^ ^330^ ^]^ SMAD7 inhibits TGF‐β signaling by blocking receptor activity, promoting receptor degradation.

#### 4.1.2 Noncanonical Signaling Pathway

In addition to the SMAD‐dependent signaling pathway, TGF‐β signaling is also activated through noncanonical signaling, such as phosphorylation, acetylation, sumoylation, ubiquitination, and protein‐protein crosstalk^[^ ^331^ ^]^ (Figure 8B). The mitogen‐activated protein kinase (MAPK) family, which regulates nuclear transcription factors through transmitting signals to intracellular targets, is an SMAD‐independent signaling pathway.^[^ ^332^ ^]^ The extracellular signal‐regulated kinases 1/2 (ERK1/2), p38/MAP kinases, and stress‐activated protein kinases such as c‐Jun amino‐terminal kinases (Jnk1/2/3) are three subfamilies of MAPKs activated by TGF‐β signaling.^[^ ^333^ ^]^ In addition, the Rho‐like signaling pathway and phosphatidylinositol‐3‐kinase (PI3K)/AKT signaling can also be activated by TGF‐β signaling.^[^ ^334^ ^]^ The PI3K/AKT signaling pathway induces profibrotic signaling, including AKT/mammalian target of rapamycin (mTOR) and p21‐activated kinase 2 (PAK2)/Abelson kinase (c‐Abl).^[^ ^335^, ^336^ ^]^

### 4.2 IL‐11 Signaling Pathway

**Figure 9:**
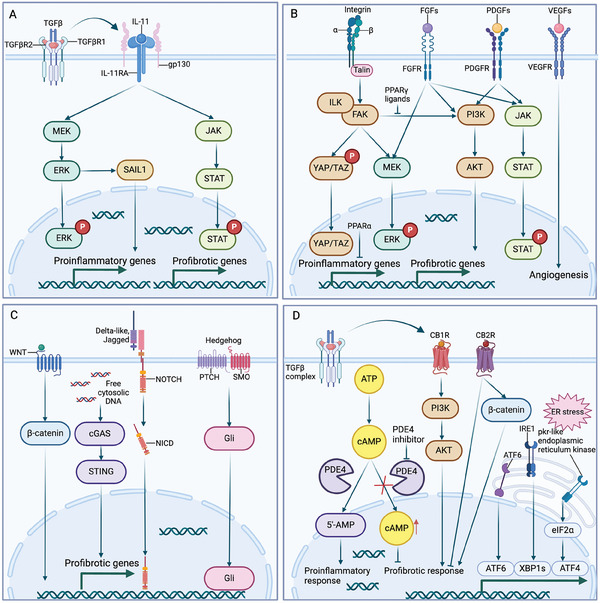
Overview of important signaling pathways involved in fibrosis. Created with Biorender.com.

IL‐11, a member of the IL‐6 family of cytokines, plays an important role in fibrosis. IL‐6 family of cytokines have one critical broadly expressed cell membrane receptor, gp130, that binds to the IL‐11 receptor α subunit (IL‐11RA) for signal transduction.^[^ ^296^, ^337^ ^]^ IL‐6 is expressed primarily in immunocytes, whereas IL‐11 is expressed mainly in stromal cells and epithelial cells. JAK/STAT3, MAPK, and AKT are downstream signals of IL‐11 in different cell types^[^ ^338^ ^]^ (**Figure** **9**A).

Studies have revealed that early STAT3 signal transduction induced by IL‐11 mediates inflammation by releasing IL‐33 and CCL20, whereas ERK signaling leads to organ fibrosis.^[^ ^339^, ^340^ ^]^ In cardiac fibroblasts, TGF‐β1 induces the upregulation of IL‐11 to promote cardiac fibrosis.^[^ ^296^ ^]^ IL‐11 binds to IL‐11RA in fibroblasts and activates noncanonical, ERK signaling pathway to trigger cardiac fibrosis. IL‐11 signaling also functions in pulmonary fibrosis. In lung fibroblasts, upregulated IL‐11 promotes the transdifferentiation of myofibroblasts in an ERK dependent, post‐transcriptional manner. A specific neutralizing antibody targeting IL‐11 blocked pulmonary fibrosis in a mouse model.^[^ ^341^ ^]^ In addition, IL‐11 is a constitute of SASP that occurs in a paracrine manner that can lead to cellular senescence, and senescent cell also secrete TGF‐β and IL‐11.^[^ ^342^ ^]^ Recently, a platform was developed to synthesize inhaled nanoparticles specifically for IL‐11 single‐chain fragment variable delivery in the lungs to suppress pulmonary fibrosis.^[^ ^343^ ^]^ One study demonstrated that IL‐11 is upregulated in A549 cells expressing the individual accessory proteins open reading frame 6 (ORF6), ORF8, ORF9b, or ORF9c from SARS‐CoV‐2.^[^ ^344^ ^]^ These ORFs stimulate the secretion of IL‐11, which promotes pulmonary fibrosis. Pathogenic IL‐11 signaling also plays an important role in alcoholic liver disease.^[^ ^345^ ^]^ TGF‐β‐activated HSCs lead to the upregulation of IL‐11, resulting in a profibrotic response through ERK signaling.^[^ ^346^ ^]^ In renal fibrosis, aberrant expression of IL‐11 is closely associated with the EMT of TECs via STAT3 and ERK signaling.^[^ ^347^ ^]^ In this study, knockout of IL‐11 significantly alleviated renal fibrosis in a UUO mouse model. In an acute kidney injury mouse model, the upregulation of IL‐11 in TECs led to SNAIL expression and renal dysfunction, resulting in renal fibrosis.^[^ ^348^ ^]^ Interestingly, Srinivas Allanki et al. revealed that IL‐11 signaling drives the cellular reprogramming that antagonizes adult mammalian scarring.^[^ ^349^ ^]^ After injury, IL‐11 in endothelial cells blocks TGF‐β signaling and EndoMT, which suppresses scarring and cardiomyocyte repopulation. Similar results revealed that the application of recombinant IL‐11 is cardioprotective and antifibrotic.^[^ ^350^, ^351^ ^]^ This contradiction could be explained by the different cell types and animal models used. Most studies have focused on the mechanisms of IL‐11/IL‐11RA in fibroblasts and epithelial cells in humans and mice. However, this study investigated IL‐11/STAT3 signaling in zebrafish endothelial cells in the heart regeneration. Mammals mainly undergo scarring in response to injury whereas regenerative species only undergo restricted scarring.

### 4.3 PDGF/PDGFR Signaling Pathway

PDGFs, which belong to a family of growth factors, are important regulators of angiogenesis, cell migration, division, growth, and proliferation.^[^ ^352^ ^]^ There are four secreted extracellular ligands encoded by four different genes and five dimeric isoforms in PDGFs, PDGF‐AA, PDGF‐BB, PDGF‐CC, PDGF‐DD, and PDGF‐AB, which are assembled into disulfide‐bonded homodimers and heterodimers.^[^ ^353^ ^]^ PDGF ligands exert their biological function by binding to PDGFRα and PDGFRβ. Then, PDGF subtypes initiate phosphorylation and activate downstream signaling cascades, such as the PI3K/AKT, JAK/STAT, and MAPK signaling pathways^[^ ^354^ ^]^ (Figure 9B). The PDGF/PDGFR signaling pathway plays an important role in fibrotic diseases. The PDGF‐BB subtype increases the expression of PDGFRβ, enhancing vascular abnormalities and the transition from pericytes to myofibroblasts in ischemia‐reperfusion‐induced renal injury mice.^[^ ^355^ ^]^ The inhibition of PDGF‐C/PDGFRα alleviates renal interstitial fibrosis.^[^ ^356^, ^357^ ^]^ PDGF‐B/PDGFRβ promotes the transdifferentiation and excessive proliferation of HSCs, which triggers hepatic fibrosis.^[^ ^358^ ^]^ In addition to PDGF‐B, PDGF‐D is also a potent PDGF isoform that contributes to hepatic fibrosis, but the effect of PDGF‐C is minimal.^[^ ^359^ ^]^ In addition, exogenous thymosin β4 attenuates cholestatic hepatic fibrosis by downregulating the PDGF/PDGFR signaling pathway.^[^ ^360^ ^]^ Nintedanib inhibits the overexpression of PDGF‐BB and alleviates pulmonary fibrosis.^[^ ^361^ ^]^

### 4.4 FGF/FGF receptor (FGFR) Signaling Pathway

The FGF/FGFR signaling pathway is involved in multiple diseases, such as obesity, insulin resistance, tumor behavior, and CKD.^[^ ^362^ ^]^ The FGF superfamily is a large cytokine family with 22 identified FGF ligands. The 18 canonical mammalian FGFs are divided into five paracrine subgroups and one endocrine subgroup on the basis of sequence homology and phylogeny.^[^ ^363^ ^]^ The five paracrine subgroups include FGF1 and FGF2; FGF4, FGF5, and FGF6; FGF3, FGF7, FGF10, and FGF22; FGF8, FGF17, and FGF18; FGF9, FGF16, and FGF20. One endocrine subgroup contains FGF19, FGF21 and FGF23.^[^ ^363^ ^]^ FGFs exert their biological functions by binding to tyrosine kinase receptors (FGFR1, FGFR2, FGFR3, and FGFR4).^[^ ^364^ ^]^ FGFRs are single‐pass transmembrane proteins that mainly consist of an intracellular tyrosine kinase domain, an extracellular domain, and a transmembrane domain. The Klotho protein is a prerequisite for the interaction between FGFs and FGFRs. α‐Klotho is essential for FGF23 activation, while β‐Klotho binds to FGF19 and FGF21 to exert their functions.^[^ ^365^ ^]^ The binding between FGFs and FGFRs induces conformational changes in FGFRs, leading to the phosphorylation of the tyrosine residues of FGFRs and the activation of downstream signaling pathways, including the Ras/Raf‐MEK‐MAPK signaling, PI3K/AKT signaling, and JAK/STAT signaling pathways^[^ ^366^ ^]^ (Figure 9B). An abnormal increase in FGF1/FGFR signaling in the lungs of IPF patients contributes to pulmonary fibrosis by supporting the migration of fibroblasts.^[^ ^367^ ^]^ FGF1 and FGF2 are associated with chemotaxis and renal interstitial fibrosis,^[^ ^368^ ^]^ and specific knockdown of early growth response 1 (Egr1) decreases the expression of FGF2 through AMPK/ERK signaling in renal TECs, which reduces the paracrine effects on fibroblasts.^[^ ^369^ ^]^ Cardiac *Fgf13* knockdown can prevent heart fibrosis induced by hemodynamic stress by regulating microtube stabilization and the ROCK signaling pathway.^[^ ^370^ ^]^ FGF21 plays important roles in glucose metabolism, lipid metabolism, energy balance, etc.^[^ ^371^ ^]^ Hepatogenic FGF21 is the major source of circulating FGF21. In addition, FGF21 is a biomarker of liver damage, as well as a therapeutic target for NAFLD. However, an increase in FGF21 is negatively associated with liver stiffness in patients infected with HCV who are receiving direct‐acting antiviral treatment.^[^ ^372^ ^]^

### 4.5 Integrin‐Linked Kinase Signaling Pathway

Emerging evidence has demonstrated that the synthesis and assembly of ECM proteins in fibrosis are determined by integrin signaling. Integrins organize the cytoskeleton and trigger intracellular signaling, regulating cell proliferation, survival, and migration.^[^ ^373^, ^374^ ^]^ The integrin family is composed of 24 heterodimeric receptors with α and β subunits, which regulate multiple ECM components.^[^ ^375^ ^]^ Upon binding to the ectodomain, activated integrins recruit the cytoskeletal protein talin to the intracellular β subunit of integrin. Integrins activate downstream kinases, FAKs, and integrin‐linked kinases (ILKs) via talin^[^ ^376^, ^377^ ^]^ (Figure 9B). ILK mediates multiple signaling pathways in fibrosis. The expression of ILK is increased in peritoneal fibrosis, and the ILK in the effluent of peritoneal dialysis initiates peritoneal fibrogenesis.^[^ ^378^ ^]^ ILK also mediates the activation of EMT in renal fibrosis and HSCs and involved in IPF in a tissue‐specific manner with a specific subset of fibroblasts expressing ILK.^[^ ^379^ ^]^ In a renal fibrosis mouse model, ILK is strongly induced and is associated with the EMT of TECs.^[^ ^380^ ^]^ In addition, angiotensin II induces the expression of TGF‐β/SMAD signaling, which promotes ILK expression.^[^ ^333^ ^]^

### 4.6 Janus kinase (JAK)/STAT Signaling Pathway

The JAK/STAT signaling pathway is critical for cytokine and growth hormone receptor signaling (Figure 9B). In mammals, JAKs are nonreceptor tyrosine kinases, including JAK1, JAK2, JAK3, and TYK2.^[^ ^381^ ^]^ JAK inhibitors were initially approved for the treatment of bone marrow fibrosis and some inflammatory diseases, such as rheumatoid arthritis and ulcerative colitis.^[^ ^382^ ^]^ FERM, SH2, pseudokinase, and kinase are four functional domains of FAK.^[^ ^383^ ^]^ Kinase is the most important domain that functions in the phosphorylation of STAT, which are translocated into the nucleus to regulate the expression of downstream genes.^[^ ^384^ ^]^ The STAT family has seven subtypes, including STAT1, STAT2, STAT3, STAT4, STAT5a, STAT5b, and STAT6.^[^ ^385^ ^]^ The interaction between cytokines and their corresponding transmembrane receptors (type 1 and type 2) is the major trigger of the JAK/STAT signaling cascade, which activates intracellular signal transduction to regulate the cell phenotype. Multiple cytokines and growth factors can activate JAK/STAT signaling, such as the IFN family, the IL‐10 family, and the PDGF family.^[^ ^386^, ^387^ ^]^ For example, cytokines and growth factors trigger the JAK/STAT signaling pathway to activate HSCs and hepatocytes, thereby inducing hepatic fibrosis.^[^ ^388^, ^389^ ^]^ In renal fibrosis, STAT3 is activated in tubulointerstitial cells, renal TECs and macrophages.^[^ ^390^ ^]^ In addition, Yangqing Chenfei formula (CYF) attenuates pulmonary fibrosis by targeting the JAK/STAT signaling pathway.^[^ ^391^ ^]^

### 4.7 PI3K/AKT Signaling Pathway

PI3K/AKT signaling is a major pathway that regulates cell growth, proliferation, motility, and metabolism^[^ ^392^ ^]^ (Figure 9B). The binding between Ras and p100 activates PI3K. In turn, the activation of receptor tyrosine kinase (RTK) triggers Ras, thereby activating PI3K.^[^ ^393^ ^]^ PI3K responds to the Toll‐like receptor signaling pathway, B‐cell receptor signaling pathway, JNK/STAT signaling pathway, and chemokine pathway.^[^ ^394^, ^395^, ^396^ ^]^ AKT is a common downstream target of PI3K and functions in the phosphorylation of multiple molecules related to cell proliferation, autophagy, and motility.^[^ ^397^ ^]^ mTOR is a direct substrate for AKT.^[^ ^398^ ^]^ PI3K/AKT signaling is involved in fibrogenic processes. The PI3K/AKT signaling pathway is a major regulator of IPF and is a potential therapeutic target for IPF.^[^ ^399^ ^]^ PI3K/AKT is activated by FTO/RUNX1, thus inducing renal fibrosis.^[^ ^400^ ^]^ In addition, PI3K/AKT can directly or indirectly regulate cardiac fibroblasts and/or the ECM to affect the progression of cardiac fibrosis.^[^ ^398^ ^]^

### 4.8 Vascular Endothelial Growth Factor (VEGF)/VEGF Receptor (VEGFR) Signaling Pathway

VEGF, which was identified more than 30 years ago, plays an important role in vasculogenesis and angiogenesis^[^ ^401^ ^]^ (Figure 9B). There are six members of the VEGF family, including VEGF‐A, VEGF‐B, VEGF‐C, VEGF‐D, VEGF‐E, and placental growth factor.^[^ ^402^ ^]^ Although several VEGF family members target endothelial cells, most studies have focused on VEGF‐A due to its critical function in regulating angiogenesis during homeostasis and disease.^[^ ^403^ ^]^ VEGF‐A binds to tyrosine kinase VEGFRs, especially the main signaling receptor VEGFR2, to exert biological functions.^[^ ^404^ ^]^ The role of VEGF‐A in pulmonary fibrosis is controversial. Several studies identified reduced expression of VEGF‐A in IPF patients.^[^ ^405^ ^]^ In a lung fibrosis mouse model, the overexpression of VEGF‐A in the lung alleviated pulmonary fibrosis.^[^ ^406^ ^]^ However, another study revealed that VEGF‐A was increased in a pulmonary fibrosis mouse model and that the inhibition of VEGF attenuated fibrosis.^[^ ^407^ ^]^ The various functions of VEGF‐A isoforms, which involve the splicing of exons, may be the reason for the dual role of VEGF‐A in pulmonary fibrosis.^[^ ^408^ ^]^ In addition, long‐term exposure to environmental pollutants promotes the production of VEGF in HSCs, thereby inducing hepatic fibrosis.^[^ ^409^ ^]^

### 4.9 YAP/TAZ Signaling Pathway

Yes‐associated protein (YAP) and transcriptional coactivator with PDZ‐binding motif (TAZ) are homologous transcriptional coactivators that act as key downstream effectors of HIPPO signaling, mechanical stimuli, and metabolism signaling. There are two main isoforms of YAP: YAP1, with one WW domain, and YAP2, with two WW domains.^[^ ^410^ ^]^ The most remarkable feature of YAP/TAZ is their ability to control overgrowth in organ size.^[^ ^411^ ^]^ When YAP/TAZ is activated, YAP/TAZ dephosphorylates specific serine residues and shuttle into the nucleus, where it binds to transcription factors (e.g., TEAD and RUNX) to regulate the expression of target genes that determine cell proliferation, survival, differentiation, homeostasis, and metabolism^[^ ^412^ ^]^ (Figure 9B). YAP/TAZ is important mediator of HIPPO signaling, WNT signaling, G‐coupled receptor signaling, and estrogen signaling.^[^ ^413^ ^]^ Notably, YAP/TAZ are also critical effectors of mechanical cues, such as ECM stiffness, stretching force, and focal adhesions.^[^ ^5^ ^]^ Interestingly, the response of YAP/TAZ to mechanical cues is independent of HIPPO/LATS and instead relies on Rho activity and the actomyosin cytoskeleton.^[^ ^414^ ^]^ YAP/TAZ is also engaged in fibrogenesis. YAP/TAZ triggers catabolism of glutamine and serine to maintain proline and glycine anabolism and promote collagen synthesis.^[^ ^415^ ^]^ In the lung, AMPK inactivation in endothelial cells activates the expression of YAP, thereby inducing pulmonary fibrosis.^[^ ^416^ ^]^ Researchers have revealed that dihydrotanshinone I (DHI), a lipophilic component from natural herbs, alleviates hepatic fibrosis via the inhibition of YAP/TEAD2.^[^ ^417^ ^]^ Gli^+^ cell‐specific deletion of *Yap*/*Taz* in a UUO mouse model resulted in decreased ECM accumulation, myofibroblast recruitment, and interstitial fibrosis.^[^ ^418^ ^]^

### 4.10 PPAR Signaling Pathway

PPARs are typical fatty acid‐activated nuclear receptors consisting of three subtypes: PPARα, PPARγ, and PPARδ (also known as PPARβ).^[^ ^419^, ^420^ ^]^ PPARα and PPARγ are highly expressed in oxidative tissues and play critical roles in substrate delivery, substrate oxidation, and oxidative phosphorylation.^[^ ^421^ ^]^ PPARα distributes mainly in the liver, brown adipose tissue, kidney and heart, and is important for fatty acid metabolism.^[^ ^422^ ^]^ PPARδ/β is expressed broadly and functions in fatty acid oxidation and blood glucose regulation. However, PPARγ, which is also expressed in the liver and skeletal muscles, is mainly responsible for energy storage by synthesizing lipids.^[^ ^423^ ^]^ In hepatic fibrosis, PPARγ is required for inactivation of HSCs and regression of hepatic fibrosis (Figure 9B). Upregulation of PPARγ inhibits TGF‐β1/SMAD signaling and blocks HSC activation.^[^ ^424^ ^]^ In addition, PPARγ can inactivate the NF‐κB signaling pathway and reduce the expression of TNFα and IL‐1β in monocyte/macrophage, thereby blocking the inflammatory response.^[^ ^425^, ^426^ ^]^ Treatment with PPARα ligand alleviates hepatic fibrosis in thioacetamide‐treated rat models of hepatic fibrosis.^[^ ^427^ ^]^ The activation of PPARα/γ attenuates insulin resistance and dyslipidemia in NAFLD, thus preventing inflammation and the progression of hepatic fibrosis.^[^ ^428^ ^]^ PPARδ/β prevents liver toxicity induced by azoxymethane and carbon tetrachloride.^[^ ^429^ ^]^ Moreover, the activation of PPARα maintains cardiac mitochondrial stability and suppresses the accumulation of cardiac lipids and fibrosis.^[^ ^430^ ^]^ A PPARγ agonist was found to inhibit TGF‐β and alleviate renal fibrosis in a UUO mouse model.^[^ ^431^ ^]^

### 4.11 Canonical WNT/β‐Catenin Signaling Pathway

The canonical WNT/β‐catenin signaling pathway regulates the nuclear translocation of β‐catenin through β‐catenin‐T‐cell factor/lymphoid enhancer‐binding factor (TCF/LEF),^[^ ^432^ ^]^ which mainly controls cell proliferation (Figure 9C). WNT signaling is highly conserved and is activated by binding between WNT ligands and membrane receptors and is responsible for morphogenesis and the organization of tissues during embryogenesis. In general, WNT/β‐catenin is suppressed in adulthood and can be reactivated in response to injury.^[^ ^433^ ^]^ WNT/β‐catenin‐dependent signaling was activated in a UUO mouse model.^[^ ^434^ ^]^ Cannabinoid receptor type 2 (CB2R) triggers the nuclear translocation of β‐catenin, which leads to renal fibrosis.^[^ ^435^ ^]^ In addition, canonical WNT signaling induces hepatic fibrosis through HSC glycolysis.^[^ ^436^, ^437^ ^]^ Cardiac injury is WNT responsive and WNT1 activates cardiac fibroblasts, inducing cardiac fibrosis.^[^ ^438^, ^439^ ^]^

### 4.12 Sonic Hedgehog Signaling Pathway

The hedgehog family is composed of sonic hedgehog (Shh), desert hedgehog (Dhh), and Indian hedgehog (Ihh).^[^ ^440^ ^]^ The Shh signaling pathway consists of the signaling molecules hedgehog, glioma‐associated oncogene homolog (Gli), patch receptor Patched (Ptch), and Smoothened (Smo), which play pivotal roles in embryonic growth, tissue patterning, cell growth and differentiation^[^ ^441^, ^442^ ^]^ (Figure 9C). Hedgehog is expressed at low levels in normal kidneys, whereas increased Shh is found in fibrotic kidneys.^[^ ^443^ ^]^ Hedgehog can induce the proliferation and transdifferentiation of interstitial fibroblasts. However, another study did not report similar results in a UUO mouse model.^[^ ^444^ ^]^ Similarly, hedgehog ligands are not expressed in normal liver tissue.^[^ ^445^ ^]^ Injury‐induced cytokines activate the secretion of Hedgehog ligands in HSCs, which are differentiated into myofibroblasts.^[^ ^446^ ^]^ Inhibition of the Shh signaling pathway markedly suppresses HSC activation and ECM synthesis, thus alleviating hepatic fibrosis.^[^ ^447^ ^]^

### 4.13 NOTCH Signaling Pathway

NOTCH signaling is highly conserved and is involved in multiple biological processes, including organ development and tissue repair^[^ ^448^ ^]^ (Figure 9C). Unlike classical pathways, the NOTCH signaling pathway is transduced independent of intermediates between membranous receptors and nuclear effectors, such as G protein‐coupled receptors (GPCRs) and enzyme‐linked receptors.^[^ ^449^, ^450^, ^451^, ^452^ ^]^ There are four paralogs of NOTCH: NOTCH1, NOTCH2, NOTCH3, and NOTCH4.^[^ ^451^ ^]^ Importantly, glycosylation of NOTCH plays a critical role in its function and stability, and changes in glycosylation enzymes largely affect the activity of NOTCH signaling in the ER.^[^ ^453^, ^454^ ^]^ The canonical and noncanonical NOTCH signaling pathways are two general NOTCH signaling pathways. In the canonical NOTCH signaling pathway, endocytosis is initiated by binding between ligands and the extracellular domains of NOTCH receptors. The NOTCH intracellular domain (NICD) is degraded in the cytoplasm or transported into the nucleus to activate the transcription of downstream genes.^[^ ^455^ ^]^ Signaling initiated by other NOTCH signaling pathways is classified as the noncanonical NOTCH signaling pathway. Researchers have profiled the transcriptomes of more than 100,000 human single cells in healthy and cirrhotic human livers, revealing several profibrotic pathways, including the PDGFR and NOTCH signaling pathways.^[^ ^456^ ^]^ NOTCH1 is also a major regulator of AEC2 fate in IPF, which contributes to the proliferation of alveolar epithelial cells and promotes pulmonary fibrosis.^[^ ^457^ ^]^ In addition, DNA methylation induces the ablation of HOXA5, thereby promoting renal fibrosis by activating the NOTCH signaling pathway.^[^ ^458^ ^]^

### 4.14 Cyclic Guanosine Monophosphate‐adenosine Monophosphate Synthase (cGAS)/ Stimulator of Interferon Genes (STING) Signaling Pathway

cGAS, which can sense pathogenic DNA and mitochondria, constitutes a surveillance system against tissue damage, is a primary cytosolic DNA sensor that binds to STING on the ER, initiating the expression of IFN‐stimulated genes that function in the canonical cGAS/STING signaling pathway^[^ ^459^ ^]^ (Figure 9C). Noncanonical signaling downstream of STING includes STING/PERK, NF‐κB, and IFN regulatory factor 3 (IRF3).^[^ ^460^ ^]^ STING functions in cellular senescence, autophagy, proliferation, and death.^[^ ^461^, ^462^, ^463^ ^]^ cGAS/STING signaling is a key crossroad between the immune system and multiple diseases, such as infection, fibrosis, and malignancy. Noncanonical STING/PERK/eukaryotic initiation factor 2α (eIF2α) signaling drives pulmonary and renal fibrosis, and is a potential therapeutic target.^[^ ^464^, ^465^ ^]^ The activation of cGAS/STING signaling promotes hepatic fibrosis and the development of hepatic sinusoidal microthrombosis.^[^ ^466^ ^]^ Oxidative stress‐mediated ferroptosis in hepatocytes regulates STING activation in macrophages, leading to hepatic injury and fibrosis.^[^ ^467^ ^]^

### 4.15 ER Stress Signaling Pathway

ER, one of the largest organelles in eukaryotic cells, functions in maintaining protein homeostasis, including protein synthesis, folding and post‐translational modification.^[^ ^468^ ^]^ ER stress occurs when protein processing is disrupted, resulting in the accumulation of unfolded or misfolded proteins in the ER. To alleviate such circumstances, ER stress triggers an unfolded protein response (UPR), an intracellular signaling network that restores homeostasis^[^ ^469^ ^]^ (Figure 9D). The UPR is mediated by activating transcription factor 6 (ATF6), inositol‐requiring enzyme 1α (IRE1α) and pkr‐like endoplasmic reticulum kinase.^[^ ^470^, ^471^ ^]^ However, long‐term high ER stress levels result in a terminal UPR program that drives the self‐destruction of cells.^[^ ^472^ ^]^

ER stress and UPR signaling are involved in fibrosis through the induction of cell death, myofibroblast transdifferentiation, EMT, and inflammatory responses.^[^ ^473^, ^474^, ^475^ ^]^ ER stress has been found in patients with pulmonary fibrosis.^[^ ^476^ ^]^ Several aetiologies, such as viral infection, aging, and amiodarone, contribute to UPR activation in the lung.^[^ ^477^, ^478^ ^]^ Some disease‐related mutations, such as surfactant protein 2A (*SFTPA2*) and *SFTPC*, are associated with IPF.^[^ ^479^, ^480^, ^481^ ^]^ UPR activation was found in AEC2s with SFTPC mutation in patients with pulmonary fibrosis. In addition, ER stress drives EMT of AECs in the lung, contributing to pulmonary fibrosis.^[^ ^482^ ^]^ Uromodulin is a plasma membrane protein that is selectively expresses in the thick ascending limb of Henle's loop in the kidney. Uromodulin gene mutations are unfolded in the ER and cause ER stress, resulting in kidney disease.^[^ ^483^ ^]^ Moreover, NLRP3, a critical component of the innate immune system, was reported to be activated by ER stress and promotes pyroptosis, apoptosis, mitochondrial damage and renal fibrosis.^[^ ^484^ ^]^ Additionally, ER stress becomes chronic in NAFLD, and UPR is activated in obese animal models.^[^ ^485^, ^486^ ^]^ Alterations of membrane fluidity and decreases in the ER Ca^+^ content lead to ER stress.^[^ ^487^ ^]^ Another mechanism involves lipid changes that also activate UPR signaling independent of the accumulation of misfolded proteins during ER stress in hepatocytes.^[^ ^488^ ^]^ These two mechanisms can both contribute to hepatic fibrosis.

### 4.16 Phosphodiesterase 4B (PDE4B) Signaling Pathway

PDEs, a superfamily consisting of 11 gene families, are enzymes that catalyze the hydrolysis of the second messengers cAMP and cyclic guanosine monophosphate (cGMP) to 5’‐AMP and 5’‐GMP, respectively^[^ ^489^ ^]^ (Figure 9D). The intracellular concentrations of cAMP and cGMP are regulated by PDEs, which also modulate PDE‐associated signaling pathways. The distribution of the PDE family is ubiquitous but varies across different cell types. The PDE4 family includes PDE4A, PDE4B, PDE4C and PDE4D, all of which catalyze the hydrolysis of Camp.^[^ ^490^, ^491^ ^]^ PDE4B is ubiquitously distributed in the brain, lung, immunocytes, heart and skeletal muscle and is an important therapeutic target of pulmonary fibrosis.^[^ ^490^, ^492^ ^]^ Preferential inhibition of PDE4B has the potential to suppress inflammation and pulmonary fibrosis.^[^ ^493^ ^]^ Inhibition of PDE4B may increase intracellular cAMP levels and suppress fibroblast proliferation, myofibroblast transdifferentiation, and ECM synthesis in the lung.^[^ ^494^ ^]^

### 4.17 Cannabinoid (CB) Signaling Pathway

The endocannabinoid system, which comprises CB receptors (CBRs), endogenous CB ligands and their biosynthetic and degradative enzymes, plays an important role in various pathophysiological processes. CBR is a G‐coupled seven‐transmembrane receptor in the CB signaling pathway. CB1R, which is expressed mainly in the central nervous system, regulates the psychoactive properties of marijuana.^[^ ^495^ ^]^ CB2R are distributed mainly in immunocytes.^[^ ^496^, ^497^ ^]^ Both CB1R and CB2R are expressed in the kidney, liver, and lung.^[^ ^498^ ^]^ CB1R signaling activation drives oxidative stress, inflammation and fibrosis, whereas CB2R signaling functions in anti‐inflammatory, antioxidative, and antifibrotic responses^[^ ^499^ ^]^ (Figure 9D). CB1R, as well as endogenous CB1 ligand, is highly expressed in both UUO‐induced renal fibrosis and in kidney biopsies of patients with IgA nephropathy, acute interstitial nephritis, and diabetic nephropathy.^[^ ^500^ ^]^ In addition, the expression of CB1R is significantly elevated in renal myofibroblasts in response to TGF‐β, which promotes fibrosis. However, the activation of CB2Rs in the heart alleviates oxidative stress, inflammation, and cardiac fibrosis after myocardial infarction and diabetes‐induced cardiac dysfunction.^[^ ^501^, ^502^ ^]^ In hepatic fibrosis, blockade of CB2Rs is related to the proliferation of myofibroblasts and HSCs.^[^ ^503^ ^]^ CB2R also exhibit anti‐inflammatory, immunosuppressive, and antifibrotic effects in the lungs and kindeys.^[^ ^504^, ^505^ ^]^

However, the role of CB2Rs in renal fibrosis is controversial. In the kidney, CB2R is induced mainly in renal TECs. CB2 knockdown suppresses TGF‐β signaling in TECs, inhibiting renal fibrosis.^[^ ^506^ ^]^ A previous study revealed that CB2Rs promote renal fibrosis by activating the WNT/β‐catenin signaling pathway.^[^ ^435^ ^]^ Hence, the specific mechanism underlying the relationship between CB2Rs and fibrosis needs further research.

## 5 Advanced Therapeutic Strategies and Clinical Trials

### 5.1 Antifibrotic Drugs Targeting Important Signaling Pathway

Multiple drugs targeting critical signaling pathways in fibrosis are under investigation and are further evaluated in clinical trials. Here, we summarize these drugs by their targets and applications.

#### 5.1.1 Targeting TGF‐β Signaling Pathway

As a key profibrotic cytokine and major mediator of fibrosis, TGF‐β has long been considered a promising therapeutic target for various fibrosis‐related diseases. This strategy starts by inhibiting activated TGF‐β generation, such as nucleic acid drugs that block TGF‐β synthesis, and molecules targeting integrin αv/β which inhibit TGF‐β activation. Moreover, blocking TGF‐β ligand‐receptor interactions and intracellular signal transduction by either TGF‐β receptor kinase inhibitors or monoclonal antibodies represents another therapeutic strategy.^[^ ^507^ ^]^

Pirfenidone (PFD): PFD is an orally bioavailable synthetic molecule that suppresses gene transcription as well as the activity of TGF‐β.^[^ ^508^, ^509^, ^510^ ^]^ It also inhibits fibroblast proliferation and snythesis of type I and type III collagen.^[^ ^511^, ^512^, ^513^ ^]^ PFD was approved by the United States Food and Drug Administration (FDA) for IPF treatment early in 2014. Multiple clinical trials have evaluated its efficacy and safety in IPF treatment^[^ ^511^, ^514^ ^]^ (**Table** **6**). In a randomized trial, PFD with a dosage of 2,403 mg/day was found to reduce the decline of forced vital capacity (FVC) by 4.4% (95% confidence interval [CI]: 0.7% to 9.1%) compared with placebo group at week 72. In addition, fewer overall deaths (19 [6%] versus 29 [8%]) as well as deaths related to IPF (12 [3%] versus 25 [7%]) were reported in the PFD 2,403 mg/day group.^[^ ^511^ ^]^ PFD has also been examined in some specific IPF patients. A phase II trial revealed that perioperative PFD treatment reduced acute exacerbation of IPF after lung cancer surgery in IPF patients.^[^ ^514^ ^]^ In advanced IPF patients, changes in the FVC and total lung capacity (TLC) are significantly reduced six months after treatment.^[^ ^515^ ^]^ Furthermore, trials are underway to explore the efficacy of PFD in the treatment of cardiac fibrosis. In a double‐blind phase II trial (NCT02932566), PFD reduced myocardial extracellular volume (between‐group difference, −1.21%; 95% CI, −2.12 to −0.31) in patients with preserved ejection fraction (HFpEF) heart failure and myocardial fibrosis.^[^ ^516^ ^]^

**Table 6:** Drug‐ based strategies in clinical trials for fibrosis.

Targets	Drug Name	Application	Phase	Status	Sample size	Trial Information
TGF‐β signaling	Pirfenidone	IPF	II	Completed	120	NCT04396756
		myocardial fibrosis	II	Completed	129	NCT02932566
	Hydronidone	HBV associated cirrhosis	III	Recruiting	248	NCT05905172
		HBV associated cirrhosis	III	Recruiting	248	NCT05115942
	HEC‐585	IPF	II	Recruiting	270	NCT05060822
	SRN‐001	IPF	I	Active, not recruiting	24	NCT05984992
	TGF‐β1 mAb	Diabetic nephropathy	II	Terminated	417	NCT01113801
Integrin family signaling	IDL‐2965	NASH	I	Terminated	6	NCT03949530
	Bexotegrast	IPF	II	Completed	120	NCT04396756
	STX‐100	IPF	II	Terminated	106	NCT03573505
		IPF	II	Completed	41	NCT01371305
	GSK3008348	IPF	I	Completed	40	NCT02612051
		IPF	I	Terminated	8	NCT03069989
β‐catenin/cyclic AMP response‐element binding protein (CBP) signaling	PRI‐724	HCV and HBV associated cirrhosis	I and II	Completed	27	NCT03620474
FGF family (Fibroblast growth factor) signaling	Aldaferrin	NASH	II	Completed	171	NCT03912532
	Pegozafermin	NASH	II	Active, not recruiting	222	NCT04929483
	Efruxifermin	NASH	II	Completed	110	NCT03976401
	Pegbelfermin	NASH with stage III fibrosis	II	Active, not recruiting	222	NCT04929483
	BMS‐986036	Nonalcoholic Steatohepatitis	II	Completed	155	NCT03486912
		Non‐alcoholic Steatohepatitis	II	Completed	184	NCT02413372
FGFR/RTK (Fibroblast growth factor receptor/receptor tyrosine kinase) signaling	Nintedanib	ILD, IPF	III	Completed	663	NCT02999178
		IPF	III	Completed	515	NCT01335464
		IPF	III	Completed	551	NCT01335477
		Systemic Sclerosis associated interstitial lung disease	III	Completed	580	NCT02597933
	ZSP1603	IPF	I and II	Recruiting	36	NCT05119972
JAK family (Janus kinase) signaling	Itacitinb	Potential cytopenias induced by ruxolitinib	II	Active, not recruiting	18	NCT04640025
	Ruxolitinib	Myelofibrosis	III	Completed	309	NCT00952289
		Myelofibrosis	III	Completed	219	NCT00934544
	Fedratinib	ruxolitinib‐resistant or ruxolitinib‐intolerant myelofibrosis	II	Completed	97	NCT01523171
	Jaktinib	ruxolitinib‐resistant or ruxolitinib‐intolerant patients	II	Completed	51	NCT04217993
PI3K/mTOR signaling	Omipalisib (GSK2126458)	IPF	I	Completed	17	NCT01725139
	HEC‐68498	IPF	I	Completed	55	NCT03502902
Sonic hedgehog signaling	Taladegib (ENV‐101)	IPF	II	Not yet recruiting	320	NCT06422884
		IPF	II	Completed	41	NCT04968574
	Vismodegib	IPF	I	Completed	21	NCT02648048
	Sonidegib (LDE225)	Myelofibrosis	I and II	Completed	50	NCT01787552
Hormone Relaxin‐2 signaling	Serelaxin	Acute heart failure	III	Completed	6600	NCT01870778
		Acute heart failure	II and III	Completed	1161	NCT00520806
PDE4B (Phosphodiesterase 4B) signaling	BI1015550	IPF	II	Completed	147	NCT04419506
		IPF	III	Recruiting	1700	NCT06238622
IL‐11 signaling	9MW 3811	IPF	I	Recruiting	32	NCT05740475
	BI765423	IPF	I	Recruiting	40	NCT06232252
CTGF (Connective tissue growth factor) signaling	FG‐3019	IPF	I	Recruiting	21	NCT00074698
		Hepatic fibrosis	II	Terminated	114	NCT01217632
Gal‐3 (Galectin‐3) signaling	Belapectin	Hepatic cirrhosis	II	Completed	162	NCT02462967
	GB0139	IPF	II	Completed	172	NCT03832946
Thrombomodulin Alfa signaling	ART‐123	Acute exacerbation of IPF	III	Completed	74	NCT02739165

HBV, hepatitis virus B; HCV, hepatitis virus C; ILD, interstitial lung disease; IPF, idiopathic pulmonary fibrosis; JAK, Janus kinase; mAb, monoclonal antibody mTOR, mammalian target of rapamycin; NASH, nonalcoholic steatohepatitis; PI3K, phosphatidylinositol‐3‐kinase.

Hydronidone: With the adverse effects of liver function impairment, the application of PFD in hepatic fibrosis is limited. Hydronidone, a novel structural modification of PFD with the aim of reducing hepatoxicity, was developed.^[^ ^517^ ^]^ Hydronidone can suppress TGF‐β production without increasing alanine transaminase (ALT)/aspartate transaminase (AST) levels. In addition, recent research has shown that hydronidone can ameliorate hepatic fibrosis by inducing activated HSC apoptosis.^[^ ^518^ ^]^ Hydronidone presented clinically significant histological improvement of hepatic fibrosis in patients with chronic HBV‐associated hepatic fibrosis in a phase II randomized controlled trial^[^ ^517^ ^]^ and its efficacy was further evaluated in phase III trials (NCT05905172, NCT05115942).

TGF‐β1 monoclonal antibody (mAb): Inhibiting TGF‐β1 activity with a specific, humanized, neutralizing mAb was explored in a placebo‐controlled phase II trial among patients with diabetic nephropathy to attenuate progressive renal fibrosis and loss of renal function. However, the addition of a TGF‐β1 mAb to renin‐angiotensin inhibitors failed to slow disease progression.^[^ ^519^ ^]^ Nevertheless, numerous preclinical studies have been conducted in renal fibrosis animal models.

Moreover, other drugs that target TGF‐β activity are under investigation. The efficacy and safety of HEC‐585, a pyrimidine compound that has structural similarities to PFD, were assessed and compared with those of PFD in IPF patients (NCT05060822). The small interfering RNA (siRNA) SRN‐001, which targets amphiregulin, a downstream fibrosis regulator induced by TGF‐β, is currently undergoing a phase I clinical trial for IPF treatment (NCT05984992). Supplementation with TGF‐β/SMAD3‐dependent antifibrotic miRNAs, such as miR‐29 remlarsen (MRG‐201), attenuated pulmonary fibrosis in mice.^[^ ^520^ ^]^ A neutralizing TGF‐β mAb (1D11) was found to relieve tissue injury and fibrosis in a hepatic fibrosis rat model.^[^ ^521^ ^]^ Moreover, the effect of TGF‐β can also be blocked by recombinant truncated TGF‐βR2.^[^ ^522^ ^]^ The LTBP‐49247, which selectively binds to and inhibits the activation of TGF‐β1 presented by LTBPs, was shown to attenuate fibrotic progression in mice.^[^ ^523^ ^]^ By inhibiting TGF‐βR1/2 kinases, GW788388 reduces renal fibrosis and decreases the transcription of ECM deposition mediators in diabetic nephropathy mice.^[^ ^524^ ^]^ TEW‐7197, which targets TGF‐βR1 kinase, also decreases the expression of collagen, α‐SMA, fibronectin, 4‐hydroxy‐2, 3‐nonenal, and integrins in a UUO mouse model.^[^ ^525^ ^]^ RNA‐based therapies targeting the downstream transcriptomes of the TGF‐β/SMAD3 pathways have also been explored.^[^ ^526^ ^]^ The canonical signaling pathway TGF‐β1/SMAD‐targeted oligodeoxynucleotide (ODN) was shown to significantly suppress UUO‐induced renal fibrosis.^[^ ^527^ ^]^ Furthermore, by targeting the MEK1/2 pathway, trametinib attenuated collagen deposition and myofibroblast differentiation and proliferation in UUO and adenine‐fed mice.^[^ ^528^ ^]^

#### 5.1.2 Targeting Integrin Signaling Pathway

STX‐100 (BG00011): STX‐100 is an integrin αvβ6 mAb that partially inhibits TGF‐β and blocks murine pulmonary fibrosis without exacerbating inflammation.^[^ ^529^ ^]^ Multiple phase II clinical trials have been conducted to evaluate the safety and efficacy of STX‐100 in IPF patients (NCT03573505, NCT01371305).

GSK3008348: GSK3008348 is an inhaled selective small molecule αvβ6 arginine‐glycine‐aspartate (RGD) sequence‐mimetic that engages integrin αvβ6. It inhibits the activation of TGF‐β with a prolonged duration of action, and reduces collagen deposition and serum C3M levels in the lung.^[^ ^530^ ^]^ Safety, tolerability, and pharmacokinetics have been examined in phase I clinical trials (NCT02612051, NCT03069989).

IDL‐2965: IDL‐2965 is an orally applied integrin antagonist that has been studied as a potential treatment for IPF and NASH. Its safety, tolerability, and pharmacokinetics are under investigation in a phase II study (NCT03949530).

Bexotegrast (PLN‐74809): Bexotegrast inhibits the TGF‐β activation by blocking integrin αvβ6 and αvβ1 binding to the RGD sequence of latent TGF‐β, thereby preventing the activation of TGF‐β.^[^ ^529^ ^]^ In a phase II multicenter trial, bexotegrast demonstrated favorable safety and tolerability profiles and antifibrotic effects according to FVC, QLF imaging, and circulating levels of fibrosis biomarkers (NCT04396756).^[^ ^531^ ^]^

#### 5.1.3 Targeting WNT/β‐Catenin Signaling Pathway

SM04646: SM04646 is a small molecule WNT pathway inhibitor delivered as an inhaled aerosol for IPF. It can suppress TGF‐β‐induced ECM gene expression and myofibroblast differentiation. A phase II clinical trial was conducted to evaluate its safety, pharmacokinetics, pharmacodynamics, and efficacy in IPF patients despite withdrawal for business reasons (NCT03591926).

PRI‐724: PRI‐724, which modulates β‐catenin/cyclic AMP response‐element binding protein (CBP) transcription, was shown to inhibit ECM deposition and alleviate hepatic inflammation in a mouse model of HCV infection and acute liver injury.^[^ ^532^, ^533^ ^]^ The safety, tolerability, and efficacy of PRI‐724 were evaluated in phase I and IIa clinical trials. Improvements in liver stiffness, the model for end‐stage liver disease score, and the serum albumin level were observed in patients with HCV‐ and HBV‐induced liver cirrhosis (NCT03620474).^[^ ^534^, ^535^ ^]^

#### 5.1.4 Targeting FGFs and RTKs Signaling Pathway

Pegozafermin: As a biomarker of liver damage, FGF21 has been widely explored as a therapeutic target for NASH and related cirrhosis. Pegozafermin, an FGF21 analog, improved fibrosis in 22% of NASH patients in the 15 mg pegozafermin group, 26% in the 30 mg pegozafermin group, and 27% in the 44 mg pegozafermin group in a phase IIb, multicenter, double‐blind, randomized, placebo‐controlled trial.^[^ ^536^ ^]^ The long‐acting Fc‐FGF21 fusion protein efruxifermin was demonstrated to alleviate hepatic fibrosis and resolve NASH over 24 weeks in NASH patients with moderate to severe fibrosis (NCT03976401).^[^ ^537^, ^538^ ^]^

Pegbelfermin (PGBF): As a polyethylene glycol‐conjugated analog of FGF21 with an extended half‐life that enables weekly dosing, pegbelfermin was found to present better NAFLD activity scores, liver stiffness (magnetic resonance elastography) and steatosis (magnetic resonance imaging‐proton density fat fraction) improvements than the placebo control in a randomized phase II trial (NCT03486912).^[^ ^539^ ^]^ In consistency, PGBF was found to improve hepatic fibrosis and liver injury/inflammation in patients with NASH and stage I‐III fibrosis in another phase II trial (NCT02413372).^[^ ^540^ ^]^

Aldafermin: Aldafermin, the engineered FGF19 analog, acts as a potent agonist of the FGFR4 and β‐Klotho (FGFR4‐KLB) receptor. Aldafermin was demonstrated to improve hepatic fibrosis in at least one stage without worsening of NASH (NCT03912532).^[^ ^541^ ^]^

Nintedanib: Nintedanib is an RTK inhibitor that targets growth factor pathways, including FGFRs, VEGFR1, VEGFR2, VEGFR3, RET, FLT‐3, and PDGFRα and PDGFRβ, and is widely applied in fibrotic interstitial lung disease (ILD).^[^ ^542^, ^543^ ^]^ Nintedanib has been demonstrated to reduce the annual rate of decline in FVC by 107 ml compared with placebo in ILD patients in a double‐blind, placebo‐controlled, phase III trial (NCT02999178).^[^ ^542^ ^]^ In patients with IPF, nintedanib reduced the decline in FVC, which was consistent with a slowing of disease progression (NCT01335464, NCT01335477).^[^ ^544^ ^]^ In addition, in some specific types of ILD, such as ILD associated with systemic sclerosis, nintedanib significantly reduced the decline in FVC (NCT02597933).^[^ ^545^ ^]^

ZSP1603: ZSP1603 is another selective RTK inhibitor that targets PDGFRs, FGFRs, and VEGFRs. It has been shown to attenuate pulmonary injury, inflammation, and fibrosis in a rat model,^[^ ^546^ ^]^ and is under investigation in a phase Ib/IIa clinical trial (NCT05119972).

#### 5.1.5 Targeting JAK/STAT Signaling Pathway

Since JAK signaling plays a crucial role in the development of myelofibrosis, the efficacy of JAK inhibitors has been widely assessed in patients with myelofibrosis.

Ruxolitinib: Ruxolitinib is the first drug approved by FDA for the treatment of myelofibrosis. As a selective inhibitor of JAK1 and JAK2, ruxolitinib selectively inhibits the proliferation of JAK2 V617F‐driven Ba/F3 cells, leading to decreased levels of phosphorylated JAK2 and STAT5. Ruxolitinib has been shown to achieve marked and durable amelioration of splenomegaly and myelofibrosis‐related symptoms and improve overall survival in multiple randomized controlled trials in patients with myelofibrosis (NCT00952289, NCT00934544).^[^ ^547^, ^548^ ^]^

Fedratinib: The selective JAK2 inhibitor fedratinib was reported to provide clinical benefit in patients with ruxolitinib‐resistant or ruxolitinib‐intolerant myelofibrosis.^[^ ^549^ ^]^ In addition, for ruxolitinib‐resistant or ruxolitinib‐intolerant patients, pan‐JAK inhibitors of JAK1, JAK2, JAK3, and TYK2 might be another option, and phase II clinical trials are ongoing to further confirm their efficacy.^[^ ^550^, ^551^ ^]^

Itacitinb: Nevertheless, there are some potential adverse effects of blockade of JAK2‐dependent hematopoietic growth factors, such as cytopenias. Thus, inhibitors targeting JAK1 only have been assessed,^[^ ^552^ ^]^ and the effect of JAK1 inhibitor itacitinb is under investigation in a phase II clinical trial (NCT04640025).

Moreover, the application of drugs targeting JAK/STAT3 signaling has also been explored in other fibrotic diseases. The effect of S3I‐201, a STAT3 inhibitor, was assessed in UUO‐induced renal fibrosis mouse model. Administration of S3I‐201 inhibited the proliferation and induced the apoptosis of renal fibroblasts and inhibited the expression of fibronectin, α‐SMA, type I collagen, and multiple cytokines.^[^ ^553^ ^]^

#### 5.1.6 Targeting PI3K Signaling Pathway

PI3Ks and mTOR are implicated in the development of IPF and have thus become potential therapeutic targets.

Omipalisib (GSK2126458): As a potent and selective inhibitor of the PI3Kα and mTOR signaling pathways, omipalisib was initially studied in antitumor therapy^[^ ^554^ ^]^ and was later reported to attenuate fibroblast proliferation and TGF‐β‐induced collagen synthesis in primary human lung fibroblasts.^[^ ^555^ ^]^ The safety and tolerability of omipalisib have been confirmed in a phase I clinical trial (NCT01725139).^[^ ^556^ ^]^

HEC‐68498: HEC‐68498 is a class I PI3K/mTOR inhibitor that was primarily used for the treatment of solid tumors. Its application in IPF has been considered in recent research, and its safety, tolerability, and pharmacokinetics in IPF patients have been investigated in a phase I clinical trial (NCT03502902).

#### 5.1.7 Targeting Sonic Hedgehog Signaling Pathway

Taladegib (ENV‐101): Taladegib is a potent Shh signaling pathway inhibitor that binds to the human Smo receptor and competitively inhibits the binding of an Smo agonist.^[^ ^557^ ^]^ Several phase II trials investigating the safety, tolerability, and impact of ENV‐101 on lung function and key measures of fibrosis in adult patients with IPF and progressive pulmonary fibrosis are ongoing (NCT06422884, NCT04968574).

Vismodegib: Vismodegib is the first approved small molecule inhibitor of the Shh signaling pathway and is initially indicated for the treatment of adults with basal cell carcinoma.^[^ ^558^ ^]^ Clinical trials have been conducted to examine the possibility of orally used vismodegib in combination with PFD for the treatment of IPF, but further studies may be limited due to tolerability issues (NCT02648048).^[^ ^559^ ^]^

Sonidegib (LDE225): Sonidegib is an inhibitor of Shh signaling pathway that selectively targets Smo, which is approved in the United States, European Union, Switzerland, and Australia for the treatment of basal cell carcinoma.^[^ ^560^ ^]^ A phase Ib/II, open‐label study assessed the safety and efficacy of the oral combination of LDE225 and ruxolitinib in patients with myelofibrosis. However, the results revealed that the combination therapy had only a modest overall benefit compared with ruxolitinib monotherapy (NCT01787552).^[^ ^561^ ^]^

#### 5.1.8 Targeting NOTCH Signaling Pathway

DAPT: DAPT is a γ‐secretase inhibitor (GSI) that blocks NOTCH signaling pathway. DAPT significantly attenuated hepatic fibrosis and decreased the expression of Snail, vimentin, and TGF‐β1 in association with the enhanced expression of E‐cadherin in rat hepatic fibrosis models. Another study revealed that DAPT treatment suppressed EMT of rat HSCs.^[^ ^562^ ^]^ DAPT also effectively reduced cholestatic liver fibrosis in a rat model.^[^ ^563^ ^]^

RO4929097: RO4929097 is another GSI and has been tested in many clinical trials of several types of cancers.^[^ ^564^ ^]^ A recent animal study indicated that RO4929097 can alleviate hepatic fibrosis partly through its effects on hepatic cell differentiation.^[^ ^565^ ^]^ Anti‐fibrosis effect of RO4929097 has also been widely studied in retinal fibrosis.^[^ ^566^ ^]^

Avagacestat: As another NOTCH pathway inhibitor, avagacestat inhibited TGF‐β‐induced HSC activation in hepatic fibrosis. In addition, a vagacestat inhibits M1 macrophage‐driven fibroblasts activation and fibroblast‐driven M1 macrophage polarization in fibroblast and macrophage co‐culture. Avagacestat also attenuated fibrogenesis in the CCL_4_‐induced hepatic fibrosis mouse model.^[^ ^567^ ^]^

#### 5.1.9 Targeting YAP/TAZ Signaling Pathway

Directly targeting YAP/TAZ is difficult; thus, interrupting the YAP/TAZ and TEAD interactions provides another option.

Verteporfin: As an inhibitor of YAP‐TEAD and TAZ‐TEAD,^[^ ^568^, ^569^ ^]^ verteporfin is combined with PFD in lipid nanoparticles (Lip@VP), which rescued pulmonary function through the inhibition of honeycomb cyst and interstitial remodeling in an animal study.^[^ ^570^ ^]^

Dihydrexidine: Dihydrexidine inhibits the translocation of YAP by activating Gαs‐coupled dopamine receptor D1 (DRD1), thereby reversing pulmonary fibrosis in mice.^[^ ^571^ ^]^

In addition, molecules derived from natural herbs, such as dihydrotanshinone I (DHI), were found to treat liver fibrosis by targeting the YAP/TEAD2 interaction of HSCs.^[^ ^417^ ^]^ More clinical trials are warranted to further evaluate the efficacy and safety of drugs that target YAP/TAZ.

#### 5.1.10 Targeting cGAS/STING Signaling Pathway

Mn@Albumin nanoparticles: Senescence of activated HSCs and related impaired immune clearance are crucial for the progression of hepatic fibrosis. Since activation of the cGAS‐STING pathway is essential for both senescence and the innate immune response, researchers have designed a cGAS‐STING stimulator that combines prosenescence with enhanced immune clearance through targeted delivery of manganese (a cGAS‐STING stimulator) via albumin‐mediated transcytosis, specifically aimed at inducing senescence and eliminating activated HSCs in hepatic fibrosis. Significant antifibrotic effects have been proven in animal models, providing a new direction for future clinical trials.

#### 5.1.11 Other Antifibrotic Drugs

Anti‐CTGF/CCN2 antibody: CTGF is a secreted glycoprotein that play an important role in fibrosis. FG‐3019 is a fully recombinant human mAb against CTGF. The safety and efficacy of FG‐3019 in IPF patients have been assessed in a phase II clinical trial. FG‐3019 was shown to reduce the decline of predicted FVC by 60.3% at week 48. FG‐3019 also lowered the proportion of patients with disease progression (NCT01890265, NCT00074698).^[^ ^572^ ^]^ In addition, FG‐3019 has also been studied in patients with HBV‐associated hepatic fibrosis despite the trial was terminated for an unexpected prominent effect of entecavir alone (NCT01217632).

CBR Inhibitors: Intraperitoneal injections of XL‐001, an inverse agonist of CB2R, ameliorated kidney injury, fibrosis, and inflammation in mice.^[^ ^506^ ^]^ Rimonabant, another selective CB1R endocannabinoid receptor antagonist, was found to regulate macrophage infiltration and decrease MCP‐1 synthesis in mice model of renal fibrosis. Thus, CB2 might be a promising therapeutic target for renal fibrosis in the future.^[^ ^500^ ^]^

Serelaxin: Human hormone relaxin‐2 is reported to participate in organ remodeling by inducing degradation of the ECM by targeting relaxin family peptide receptors (RXFPs), which subsequently induce the production of VEGF, eNOS, and MMPs.^[^ ^573^ ^]^ Serelaxin is a recombinant form of the human hormone relaxin‐2. In cardiac fibrosis mouse models, serelaxin exerted antifibrotic effects through inhibition of EndoMT through endothelial RXFP1.^[^ ^574^ ^]^ The safety and efficacy of serelaxin in multiple cardiovascular diseases have been evaluated in clinical trials (NCT01870778 and NCT00520806).^[^ ^575^, ^576^ ^]^

PDE4B inhibitors: PDE4B inhibition is associated with anti‐inflammatory and antifibrotic properties.^[^ ^577^ ^]^ BI1015550 is an oral preferential inhibitor of PDE4B.^[^ ^578^ ^]^ A phase II clinical trial demonstrated that treatment with BI1015550, either alone or in combination with antifibrotic agents, alleviated the decrease in pulmonary function in patients with IPF (NCT04419506).^[^ ^579^ ^]^ Multiple phase III trials are ongoing to further evaluate its efficacy and safety in IPF (NCT06238622, CTR20242619).

IL‐11 antibody: IL‐11, which is secreted by fibroblasts, is upregulated in the lung of patients with IPF. Elevated IL‐11 in turn promotes lung fibroblasts transdifferentiation in an ERK‐dependent, post‐transcriptional manner.^[^ ^341^ ^]^ Multiple IL‐11 mAbs have been designed to treat IPF. 9MW 3811 is an IL‐11 antibody that was assessed in two phase I clinical trials for IPF (NTC05740475 and CTR20231721). Another antibody, BI765423, is also under investigation (NCT06232252 and NCT05658107).

Gal‐3 inhibitor: Gal‐3 is a β‐galactoside‐binding mammalian lectin and part of the 15 members in galectin family that are highly evolutionarily conserved. Gal‐3 is potently profibrotic and modulates the activity of fibroblasts and macrophages in chronically inflamed organs. Galectin inhibitors target both secreted and membrane‐associated galectins.^[^ ^580^ ^]^ Belapectin (GR‐MD‐02) is a complex carbohydrate molecule derived from a natural plant compound. It contains oligosaccharide chains containing galactose residues, binds to Gal‐3, and inhibits function of Gal‐3.^[^ ^581^ ^]^ The safety and pharmacokinetics of belapectin have been confirmed in a randomized clinical study in NASH participants with bridging fibrosis.^[^ ^582^ ^]^ However, in a phase IIb, randomized trial assessing belapectin in patients with NASH, cirrhosis, and portal hypertension, belapectin was found to be safe but not associated with significant reduction in fibrosis. Belapectin only reduces hepatic venous pressure gradient and the development of varices in patients without esophageal varices (NCT02462967).^[^ ^583^ ^]^ Another Gal‐3 inhibitor, GB0139, was studied in IPF. Inhalation of GB0139 of 3 mg/day was associated with improved respiratory‐related quality of life over 52 weeks (NCT03832946).

Thrombomodulin α (ART‐123): ART‐123 is a novel, recombinant and soluble thrombomodulin. It is a human protein with both thrombin inhibiting and protein C stimulating activities, and is applied for treatment of thromboembolism and blood clotting disorders, such as disseminated intravascular thromboembolism.^[^ ^584^ ^]^ It has also been utilized in IPF treatment. A multicenter, double‐blind, randomized, placebo‐controlled, parallel group comparison study has assessed the efficacy and safety of the intravenous drip infusion of ART‐123 in patients with acute exacerbation of IPF. However, the results showed that ART‐123 did not improve the 90‐day survival rate (NCT02739165).

### 5.2 Cellular Therapy for Fibrosis

**Figure 10:**
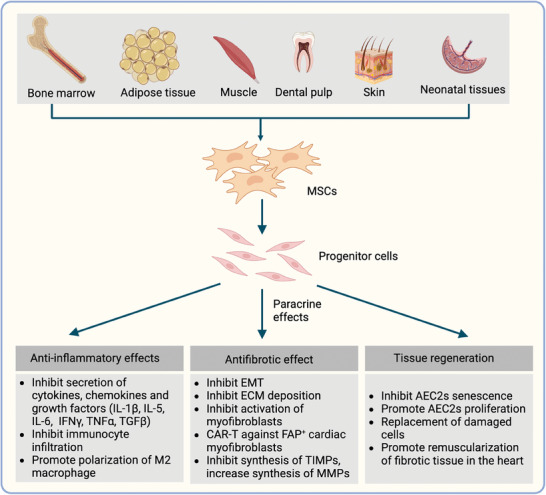
Mechanisms of mesenchymal stem cell (MSC)‐based therapy for fibrosis. The main cell sources of MSCs mainly include bone marrow, adipose tissue, muscle, dental pulp, skin, and neonatal tissues. MSCs are progenitors that function in anti‐inflammatory effects, antifibrosis, and tissue regeneration via the paracrine approach. Created with Biorender.com.

Cellular therapy, also known as cellular regenerative therapy, aims to restore impaired structure and function of living tissues and organs using cell transplantation technology. Stem cell therapies may be the most advanced therapies with high hopes in the treatment for disease that has not received ideal efficacy with conventional drugs.^[^ ^585^ ^]^ Stem cell therapy mainly includes tissue stem cells‐derived therapy and pluripotent stem cells (PSCs)‐derived therapy, including embryonic stem cells (ESCs) and induced PSCs (iPSCs). Cellular therapies are generally divided into two categories: 1) Cell infusion to the patients to activate tissue repair system through regulation of immune response or secretion of some factors and exosomes; 2) Cell‐replacement therapy (CRT) to provide cells as alternatives of senescent, injured or died cells.^[^ ^586^ ^]^ Cellular therapies, especially MSCs‐based therapy, are regarded as promising therapies for fibrosis (**Figure** **10**). Considering the immunomodulatory and regenerative potential of distinct stem cell types, transplantation of various stem cell types has been investigated in fibrotic diseases by many researchers.^[^ ^587^ ^]^ A large amount of ongoing research and clinical trials have shown the great potential of MSCs in suppressing fibrosis despite some concerns about the safety and efficacy of cellular therapies. In this section, we discuss the recent advances in research and clinical trials of cellular therapies, aiming to provide some evidence for the development of cellular therapies targeting fibrosis.

#### 5.2.1 Cellular Therapy for Renal Fibrosis

Stem cells have been widely studied in renal fibrosis. Some studies have indicated that stem cells can alleviate ECM accumulation in renal fibrosis.^[^ ^588^ ^]^ Currently, the main stem cells used in renal fibrosis are MSCs, including bone marrow‐derived MSCs (BM‐MSCs), umbilical cord‐derived MSCs (UC‐MSCs), amniotic fluid mesenchymal stem cells (AF‐MSCs), adipose‐derived MSCs (AD‐MSCs), Wharton's jelly‐derived MSCs (WJ‐MSCs), and dental mesenchymal stem cells (DMSCs). MSCs have been reported to block activation of proinflammatory cytokines and fibrosis‐associated signaling pathways, including TGF‐β/SMAD, NF‐κB, MAPK/ERK, and PI3K/AKT signaling pathway in animal studies, and consequently reduce EMT and ECM accumulation.^[^ ^588^, ^589^, ^590^ ^]^ Moreover, MSC‐derived EVs have also been widely studied and shown to participate in renal fibrosis. AD‐MSC‐derived exosomes were reported to inhibit transition of renal TECs to their profibrogenic phenotype by upregulating Sox9,^[^ ^591^ ^]^ whereas UC‐MSC‐derived exosomes attenuate renal fibrosis through CK1δ/β‐TRCP‐inhibited YAP activity.^[^ ^592^ ^]^ The safety of allogeneic MSC administration in nephrology was assessed in a phase I clinical trial in patients at high risk of developing AKI after undergoing on‐pump cardiac surgery. The results showed that postoperative infusion of allogeneic MSCs at incremental doses is safe and reduces occurrence of AKI (NCT00733876) (Figure 7). Another phase I clinical trial involving kidney allograft recipients with signs of rejection and/or an increase in interstitial fibrosis/tubular atrophy (IF/TA) was conducted. MSC infusion was reported to be well‐tolerated, safe, and resolved tubulitis without IF/TA (NCT00734396).^[^ ^593^ ^]^ However, another study reported the opposite results. A case report described a CKD patient with stable renal function for many years before MSC administration. Intravenous infusion of autologous AD‐MSCs aggravated renal insufficiency, severe interstitial fibrosis, and inflammatory cell infiltration.^[^ ^594^ ^]^ Therefore, more clinical trials are warranted to further confirm the safety and efficacy of stem cell and stem cell‐related therapies in renal fibrosis treatment (**Table** **7**).

**Table 7:** Typical MSCs‐based cellular therapies in clinical trials for fibrosis.

Origin	Application	Phase	Status	Sample size	Trial Information
Bone marrow‐derived MSCs	High risk of developing AKI after undergoing on‐pump cardiac surgery	I	Completed	15	NCT00733876
	Kidney allograft recipient	I and II	Completed	15	NCT00734396
	IPF	I and II	Completed	20	NCT02594839
	Alcoholic liver cirrhosis	II	Unknown	12	NCT01741090
Umbilical cord‐derived MSCs	Hepatic fibrosis	I and II	Completed	266	NCT01220492
Adipose‐derived MSCs	Hepatic fibrosis	I	Completed	6	NCT02297867
	IPF	I	Completed	24	NCT04313647
Immunomodulatory progenitor cells	Cardiac fibrosis	II	Unknown	50	NCT03515291
Bone marrow‐derived mononuclear cells	Hepatic fibrosis	I and II	Completed	30	NCT01120925

AKI, acute kidney injury; IPF, idiopathic pulmonary fibrosis; MSCs, mesenchymal stem cells.

#### 5.2.2 Cellular Therapy for Hepatic Fibrosis

The mechanism of stem cell therapy in hepatic fibrosis has been summarized in two major pathways: 1) Differentiation into functional cells to replace damaged cells; 2) Produce bioactive factors that modulate inflammation and fibrosis.^[^ ^595^ ^]^ MSCs are the most widely used stem cells in the treatment for hepatic fibrosis. In addition, MSCs of different origins, such as bone marrow, umbilical cord, adipose, menstrual blood, dental pulp, and liver, have different effects on the treatment.

UC‐MSCs for patients with decompensated hepatic fibrosis were assessed in a phase I/II trial (NCT01220492). Intravenous administration of UC‐MSCs significantly decreased the ascites volume and increased serum albumin level in patients and improved the model of end‐stage liver disease (MELD) scores.^[^ ^596^ ^]^ Moreover, antifibrotic effect of BM‐MSCs in alcoholic liver cirrhosis was studied in another trial. Improvements in histology and the Child–Pugh score, decreases in the levels of TGF‐β1, type I collagen, and α‐SMA were observed with BM‐MSCs injection via hepatic artery (NCT01741090). For autologous AD‐MSCs, intrahepatic injection of AD‐MSCs improved liver function, the Child–Pugh score, and the MELD score (NCT02297867). Since many factors may influence the efficacy of MSC‐based therapy, a systematic review and meta‐analysis revealed that a single injection administration via the hepatic artery and BM‐MSCs appeared to be the optimal choices to improve liver function.^[^ ^597^ ^]^ Moreover, with genome editing broadly studied, clustered regularly interspaced short palindromic repeats and the CRISPR‐associated protein 9 (CRISPR/Cas9) provide a more efficient way to optimize MSC therapy for hepatic fibrosis in the future.^[^ ^598^ ^]^ Nevertheless, long‐term efficacy of MSC therapy and detailed analyses of treatment choices in different situations warrant further investigation.

Bone marrow mononuclear cells (BMMNCs) are isolated from bone marrow and are composed of multiple stem/progenitor cells. CD11b^+^CD14^+^ BMMNCs improved hepatic fibrosis by reducing oxidative stress and inflammation in mouse models.^[^ ^599^ ^]^ One clinical trial reported that injection of BMMNCs via the portal vein in decompensated hepatic fibrosis patients, but no significant efficacy was observed (NCT01120925). In addition, bone marrow‐derived macrophages delivery improved liver function in a mouse hepatic fibrosis model. Clinical trials are needed to confirm its therapeutic potential.^[^ ^600^ ^]^

#### 5.2.3 Cellular Therapy for Pulmonary Fibrosis

Stem cells and endogenous lung progenitors have been extensively studied for their regenerative potential in chronic diseases due to their self‐renewal and multilineage differentiation capabilities, which are pivotal for tissue repair.^[^ ^601^ ^]^ In addition, recent research has shown that transplanted stem cells have paracrine effects, such as angiogenesis modulation and regulation of inflammatory and immune reactions in mouse models of lung disease.^[^ ^602^ ^]^ Stem cells used for the treatment of IPF include endogenous lung stem/progenitor cells, ESCs, iPSCs, and MSCs. MSCs are currently the most commonly used stem cells in clinical trials because of their low immunogenicity and tumorigenicity and the lack of potential ethical problems.^[^ ^603^ ^]^ The safety, tolerability and efficiency of the administration of MSCs in IPF patients have been evaluated in several clinical trials (NCT02594839).^[^ ^604^, ^605^, ^606^ ^]^ Although changes in the high‐resolution computed tomography fibrosis score were observed only in one pilot study,^[^ ^607^ ^]^ improvements in lung function and quality of life were reported in many of these trials. Nevertheless, many factors affect the efficacy of therapy including delivery route, dose of transplanted cells, administration frequency, types of MCS (BM‐ MCS, UC‐MCS, AD‐MCS, amniotic fluid stem cells, and human placental MSCs).^[^ ^608^ ^]^ Moreover, stem cell‐derived EVs were found to exert the therapeutic effects of stem cells.^[^ ^608^ ^]^ Several animal studies have demonstrated antifibrotic, anti‐inflammatory, and immunoregulatory function of EVs. The safety of nebulized human adipose‐derived MSC‐EVs (haMSC‐EVs) has also been evaluated in a phase I clinical trial (NCT04313647).^[^ ^609^ ^]^

Epithelial cells have been evaluated in multiple clinical trials for IPF. AEC2s serve as progenitor cells within the adult lung, and play a crucial role in alveolar regeneration following pulmonary injury. Intratracheal transplantation of AEC2s was found to inhibit the fibrogenic process in a rat model of pulmonary fibrosis.^[^ ^610^ ^]^ The transplantation of human AEC2s in individuals with moderate to severe IPF has been found to be safe, with good tolerance and no significant side effects in a clinical trial.^[^ ^611^ ^]^ Clinical trials further evaluating its efficacy are warranted. Moreover, since basal cells act as airway stem cells that can generate all airway epithelial cell types, basal cells have also been studied as a potential cellular strategy for treating bronchiectasis. Application of basal cells in IPF could be further estimated.^[^ ^612^ ^]^

#### 5.2.4 Cellular Therapy for Cardiac Fibrosis

BM‐MSCs are an important source of cardiac myofibroblasts. The use of stem cells as a form of cardiac therapy dates back 20 years.^[^ ^613^ ^]^ There are two main approaches to apply this therapy. Direct remuscularization of fibrotic tissue in injured heart refers to the transplantation of cells into the injured area, leading to possible integration with viable cells in the host myocardium. Potential cell types for transplantation include autologous skeletal myoblasts, bone marrow‐derived CD34^+^ cells, C‐kit surface antigen‐selected cells, ESC/iPSC‐derived cardiomyocyte precursors, and ESC/iPSC‐derived cardiac myofibroblasts.^[^ ^587^ ^]^ Another approach plans to use cells or cellular products, such as exosomes, to induce endogenous progenitors or cardiac fibroblasts to proliferate and replace fibrotic tissue in the injured myocardium.^[^ ^614^ ^]^ A previous clinical study revealed that participants who received injections of immunomodulatory progenitor cells (iMP cells, “Heartcel”) experienced a reversal of heart muscle scarring when the cells were injected into heart muscle during coronary artery bypass graft (CABG) surgery.^[^ ^615^ ^]^ A phase IIb, randomized, double‐blinded, and placebo‐controlled trial with a larger sample size was ongoing to further examine the efficacy and safety of intramyocardial injection of allogeneic human iMP cells in patients undergoing CABG surgery (NCT03515291).

After myocardial injury, cardiac myofibroblasts are major mediator cells in pathological remodeling. Therefore, reprogramming fibroblasts into induced cardiac‐like myocytes instead of myofibroblasts has become a promising method.^[^ ^87^ ^]^ Success in human fibroblast reprogramming started in 2013. Nam et al. partially converted fibroblasts into cardiac‐like myocytes via the combination of Gata4, Hand2, Tbx5, Myocd (myocardin), miR‐1 and miR‐133.^[^ ^616^ ^]^ Remarkable progress has been made especially with the application of small molecules.^[^ ^617^ ^]^ However, there is still a long way to go before its clinical application.

Since fibroblast activation protein (FAP) is overexpressed on cardiac myofibroblasts, engineered chimeric antigen receptor T cell (CAR‐T) cells against FAP may significantly reduce myocardial fibrosis and improve cardiac function in a cardiac fibrosis mouse model.^[^ ^618^ ^]^ Transient anti‐fibrotic CAR‐T cells were later generated and its efficacy in reducing fibrosis and restore cardiac function was studied in animal study.^[^ ^619^ ^]^ Nevertheless, the toxic effects of CAR‐T cells on the cardiovascular system need to be further elucidated.

## 6 Conclusion

Owing to the diversity of cell types and signaling cascades, the sophisticated pathogenesis of fibrosis presents great challenges in understanding the key mechanisms and effective therapies targeting fibrosis. Although many drugs have shown ideal antifibrotic efficacy in clinical trials, the mechanisms underlying their ability to prevent injury to normal cells is still unknown. The development of scRNA‐seq provides a detailed atlas of fibroblast subpopulations that contribute to fibrosis, which can help researchers investigate precise molecules that target these harmful cells. In addition, sensitive biomarkers to predict fibrosis in the clinic are needed. Therefore, exploration and understanding of the interactions of these mechanisms are important approaches to drugs in reversing fibrosis with restricted adverse effects.

## Conflict of Interest

The authors declare no conflict of interest.

## Author Contributions

X.D. and Y.L. contributed equally to this work. X.D. and B.L. conceived the project and constructed the outline of this manuscript. J.W. performed the outline of the history overview of fibrosis. T.L. reviewed and edited the manuscript. X.D. and Y.L. drafted the manuscript. X.D. and B.L. provided funds for this manuscript. All authors read and approved of the final manuscript.

## Data Availability

Not applicable.
